# Differences in Multicomponent Pharmacokinetics, Tissue Distribution, and Excretion of Tripterygium Glycosides Tablets in Normal and Adriamycin–Induced Nephrotic Syndrome Rat Models and Correlations With Efficacy and Hepatotoxicity

**DOI:** 10.3389/fphar.2022.910923

**Published:** 2022-06-09

**Authors:** Wei Wu, Rui Cheng, Hamza Boucetta, Lei Xu, Jing–ru Pan, Min Song, Yu–ting Lu, Tai–jun Hang

**Affiliations:** ^1^ Key Laboratory of Drug Quality Control and Pharmacovigilance (China Pharmaceutical University), Ministry of Education, Nanjing, China; ^2^ Department of Pharmaceutical Analysis, China Pharmaceutical University, Nanjing, China

**Keywords:** tripterygium glycosides tablets, nephrotic syndrome, pharmacokinetics, tissue distribution, excretions, efficacy, hepatotoxicity

## Abstract

Tripterygium glycosides tablets (TGT) are widely used for treating nephrotic syndrome (NS), but hepatotoxicity is frequently reported. The presence of underlying disease(s) can alter the disposition of drugs and affect their efficacy and toxicity. However, no studies have reported the impact of NS on the ADME profiles of TGT or its subsequent impact on the efficacy and toxicity. Thus, the efficacy and hepatotoxicity of TGT were evaluated in normal and NS rats after oral administration of TGT (10 mg/kg/day) for 4 weeks. The corresponding ADME profiles of the six key TGT components (triptolide (TPL), wilforlide A (WA), wilforgine (WFG), wilfortrine (WFT), wilfordine (WFD), and wilforine (WFR)) were also measured and compared in normal and NS rats after a single oral gavage of 10 mg/kg TGT. Canonical correlation analysis (CCA) of the severity of NS and the *in vivo* exposure of the six key TGT components was performed to screen the anti–NS and hepatotoxic material bases of TGT. Finally, the efficacy and hepatotoxicity of the target compounds were evaluated *in vitro*. The results showed that TGT decreased the NS symptoms in rats, but caused worse hepatotoxicity under the NS state. Significant differences in the ADME profiles of the six key TGT components between the normal and NS rats were as follows: higher plasma and tissue exposure, lower urinary and biliary excretion, and higher fecal excretion for NS rats. Based on CCA and *in vitro* verification, TPL, WA, WFG, WFT, WFD, and WFR were identified as the anti–NS material bases of TGT, whereas TPL, WFG, WFT, and WFD were recognized as the hepatotoxic material bases. In conclusion, NS significantly altered the ADME profiles of the six key TGT components detected in rats, which were related to the anti–NS and hepatotoxic effects of TGT. These results are useful for the rational clinical applications of TGT.

## 1 Introduction

Nephrotic syndrome (NS) is a refractory and complex chronic kidney disease (CKD) characterized by massive proteinuria, hypoalbuminemia, hyperlipidemia, and edema (Wang and Greenbaum, 2019). *Tripterygium wilfordii Hook. F* is a well–known traditional Chinese medicine (TCM) with prominent anti–inflammatory and immunosuppressive effects (Chen et al., 2013). Tripterygium glycosides tablets (TGT) formulated with only *Tripterygium wilfordii Hook. F* chloroform extract have been widely used for the treatment of NS in clinical settings for many years (Zhu et al., 2013; Wang et al., 2020). Nevertheless, TGT may also lead to adverse events, particularly hepatic injuries, due to its narrow therapeutic window (Peng et al., 2021; Zhang et al., 2021). These adverse events then restrict its clinical applicability.

**FIGURE 1:**
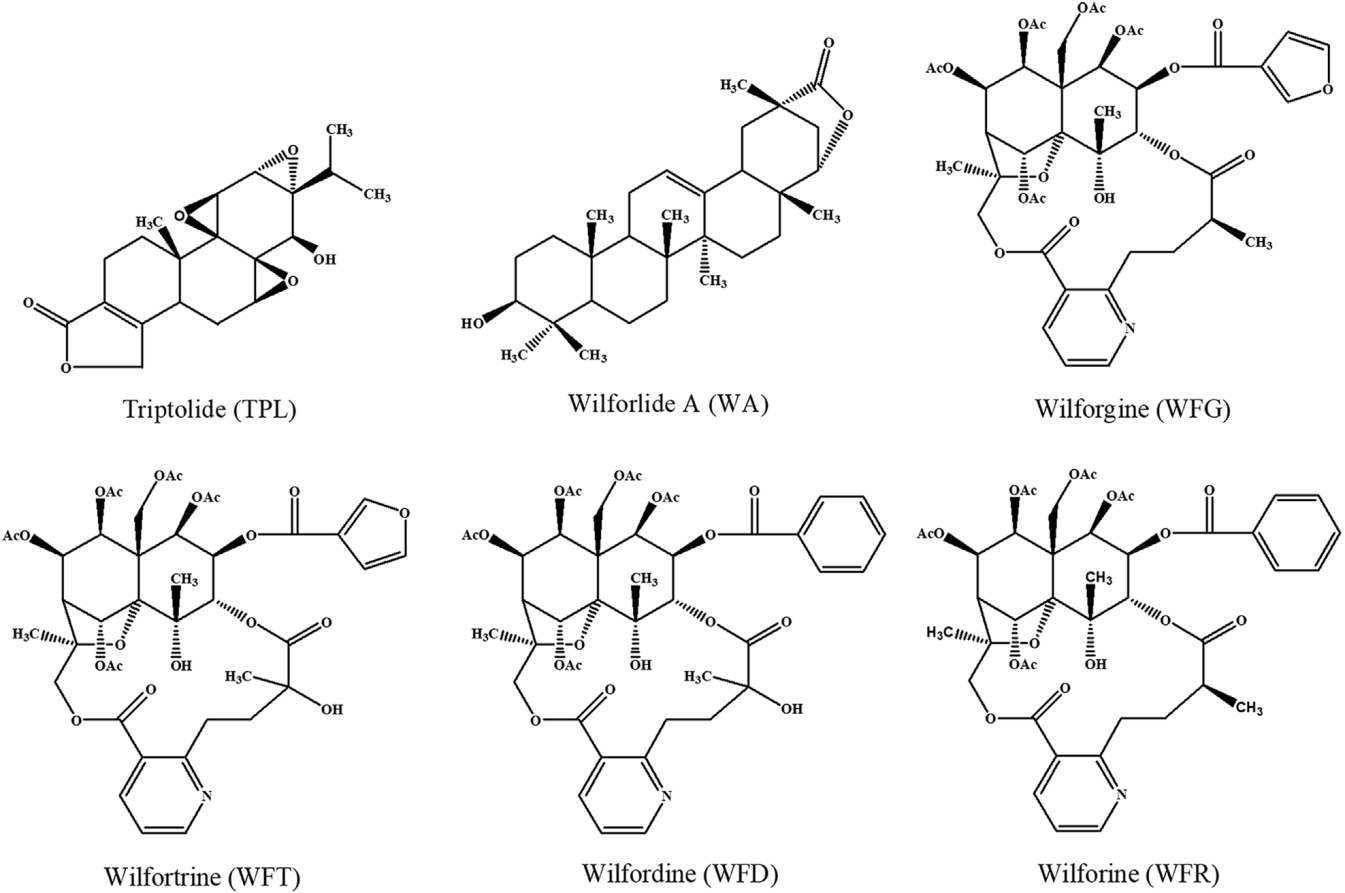
Chemical structures of the six key TGT components.

Triptolide (TPL), wilforlide A (WA), and four sesquiterpene pyridine alkaloids, wilforgine (WFG), wilfortrine (WFT), wilfordine (WFD), and wilforine (WFR) (Figure 1), have been identified as the major bioactive components of TGT (Gao et al., 2015; Wang et al., 2018; Tong et al., 2021). To ensure the safety of this medicine, the Chinese National Medical Products Administration has released the quality control standard of TGT (WS3–B–3350–98–2011), requiring that the amount of TPL in TGT should not exceed 10 μg/tablet, while that of WA should not be less than 10 μg/tablet (Chinese Pharmacopoeia Commission, 2011). A recent study suggested that WFR could also serve as a quality marker of TGT (Gao et al., 2019). Moreover, researchers have found sesquiterpene pyridine alkaloids have inhibitory effects on the production of nitric oxide and cytokines (Duan et al., 2001; Gao et al., 2015). However, growing evidence shows that these components can also cause serious toxicity. Recently, two studies reported hepatotoxic effects associated with TPL, WFG, and WFR administration in mice (Li et al., 2015; Yuan et al., 2020). Thus, subtle changes in the disposition of these bioactive ingredients *in vivo* could affect both the efficacy and toxicity of TGT, due to their narrow therapeutic windows.

Since drugs are used to treat diseases, patients are their ultimate consumers. However, the pathological status and the severity of disease will affect the drug disposition. These effects can be explained by the altered activities and expression of typical drug transporters and cytochrome P450s (CYP450s), as well as gastrointestinal lesions caused by diseases (Evers et al., 2018; Storelli et al., 2018; Vinarov et al., 2021). Previous studies have shown that CKD affects the disposition of other TCMs (Zhao et al., 2015a; Zhao et al., 2015b). Ultimately, alterations in drug disposition converge to affect clinical outcomes. In response to these changes, clinicians must adjust dosing regimens rationally based on therapeutic drug monitoring, especially for drugs with narrow therapeutic windows. Thus, to better understand the mechanism(s) of action of TGT in the treatment of CKD and support its rational use in the clinic, it is necessary to compare the ADME profiles of the six key TGT components in healthy and CKD states. Although researchers have studied the pharmacokinetic and toxicological characteristics of some TGT components (Du et al., 2018), to our knowledge no studies have reported the detailed ADME profiles of the six key TGT components after oral TGT, or performed a comparative ADME study between healthy and CKD states.

To address these issues, we comparatively investigated the ADME profiles of the six key TGT components in normal and NS rats after oral gavage of TGT using a validated UHPLC–MS/MS method for the bioassay. The therapeutic efficacy and side effects of TGT in NS rats were also evaluated. Based on the results of canonical correlation analysis (CCA) and cell experiments, the anti–NS and hepatotoxic material bases were further confirmed. These results are useful for the rational clinical applications of TGT.

## 2 Materials and Methods

### 2.1 Reagents and Materials

TPL (Lot: 111,567–202,005) and irbesartan (as the IS for the MS/MS determination, Lot: 100,607–201,804) reference substances (content >99%) were obtained from National Institute for Food and Drug Control (Beijing, China). WA (Lot: wkq20040901), WFG (Lot: wkq20040804), WFT (Lot: wkq20040203), WFD (Lot: wkq20040702), and WFR (Lot: wkq20040205) reference substances (content >98%) were purchased from Sichuan Weikeqi Biological Technology Co., Ltd. (Sichuan, China). Adriamycin (ADR) hydrochloride for injection (Lot: 2007E1) was provided by Shenzhen Main Luck Pharmaceuticals Inc. (Shenzhen, China). Assay kits for the measurement of proteinuria, blood albumin (ALB), total protein (TP), total triglycerides (TG), and total cholesterol (TC) were purchased from Nanjing Jiancheng Biological Engineering Institute (Nanjing, China). Lipopolysaccharide (LPS, *Escherichia coli* 0111: B4) was obtained from Sigma–Aldrich (St. Louis, MO, United States ). The Cell Counting Kit–8 (CCK–8) was purchased from Beyotime Institute Biotechnology (Shanghai, China). HPLC–grade methanol and acetonitrile were obtained from Tedia Company, Inc. (Fairfield, OH, United States ). Analytical grade ethyl acetate, ammonium acetate, and formic acid were supplied by Nanjing Chemical Reagent Co. Ltd. (Nanjing, China). Ultrapure water was prepared using a Millipore purification system (Millipore, MA, United States ).

TGT (Lot: 200,701, containing 10 mg *Tripterygium wilfordii Hook. F* chloroform extract per tablet), a commercially available TCM preparation, were purchased from Jiangsu Meitong Pharmaceutical Co. Ltd. (Jiangsu, China). Each tablet was measured as containing 5.90 μg TPL, 25.4 μg WA, 148 μg WFG, 86.0 μg WFT, 122 μg WFD, and 175 μg WFR, using our previously validated method (Su et al., 2015b). The content of TPL was less than 10 μg, while that of WA was more than 10 µg per tablet, which met the quality control standard for TGT, WS3-B-3350–98-2011 (Chinese Pharmacopoeia Commission, 2011).

### 2.2 UHPLC–MS/MS Conditions

A UHPLC–MS/MS system (TSQ Quantis, Thermo Scientific, San Jose, CA, United States ) equipped with an electrospray ionization interface (ESI) operating in the positive ion mode was used for analysis. Chromatographic separation was achieved on a BDS Hypersil™ C8 column (150 × 2.1 mm, 2.4 µm) with linear gradient elution at 0.5 ml/min using 10 mM ammonium acetate buffer solution and methanol containing 0.1% formic acid as the mobile phases A and B, respectively. The elution program was set as follows (A:B): 0 min (40:60) → 2 min (40:60) → 3 min (0:100) → 9 min (0:100) → 9.1 min (40:60) → 10 min (40:60). The column temperature was maintained at 40°C and the sample injection volume was 20 µl. The MS/MS conditions were optimized as follows: spray voltage of 4.5 KV; ion transfer tube temperature of 350°C; vaporizer temperature of 150°C; nitrogen sheath gas of 241 kPa; and auxiliary gas of 158 kPa. The quantification analysis was conducted under the multiple reaction monitoring (MRM) mode with the argon gas collision–induced dissociation (CID) pressure set at 0.3 Pa. The ion reactions were *m/z* 378.34@11eV → 361.10 for TPL, *m/z* 472.46@12eV → 437.30 for WA, *m/z* 858.35@55eV → 178.08 for WFG, *m/z* 874.32@24eV → 846.25 for WFT, *m/z* 884.37@24eV → 856.25 for WFD, *m/z* 868.41@55eV → 178.08 for WFR, and *m/z* 429.31@23eV → 207.00 for IS.

### 2.3 Induction and Assessment of NS Rats by Adriamycin Injection

Approximately 165 healthy male Sprague–Dawley rats (6–8 weeks, 200 ± 20 g) were obtained from Shanghai SIPPR–BK laboratory animal Co. Ltd. (Shanghai, China, SCXK (Hu) 2018–0006) and used in this study. All rats were acclimatized in the laboratory for a week and kept under specific pathogen–free conditions: 25 ± 5°C, 50 ± 20% humidity, 12/12 h light/dark cycle, with free access to food and water. All animal experiments were approved by the Animal Ethics Committee of the China Pharmaceutical University (approval number: 2021–09–007).

Since adriamycin–induced nephropathy in rats is a classical animal model of NS and the renal pathological changes are similar to those in patients with NS (Bertani et al., 1982), three types of rats (*n* = 42 in each type) were established: normal control rats (Con), early–stage NS rats (M1), and advanced–stage NS rats (M2). For rats, the optimal intravenous dose of adriamycin used to induce NS ranges from 1.5 to 7.5 mg/kg (Lee and Harris, 2011). The M1 rats were induced with a 4 mg/kg dose of adriamycin through tail vein injection on day 1 and an additional 2 mg/kg dose of adriamycin after 1 week (Li et al., 2019; Li et al., 2020), while the M2 rats were induced with a single 7.5 mg/kg intravenously dose of adriamycin (Guo et al., 2014; Ni et al., 2018). The Con rats were injected with an equal volume of sterile saline. At the end of the fourth week after the first adriamycin injection, 24 h urine samples from all rats were collected, and their volumes were recorded. After centrifugating the samples at 1,500 ×*g* for 5 min, we measured the 24 h urinary protein level to evaluate the extent of NS. Approximately 100 μL of blood from all rats was also collected in tubes without anticlotting reagents via the orbital venous plexus, and the corresponding serum samples were separated by 1,100 ×*g* centrifugation for 10 min at 4 °C for the analysis of blood biochemical indices (ALB, TP, TG, TC).

### 2.4 Dosing Strategy and Preparation of TGT Suspension

In this study, we administered oral doses of 10, 30, and 90 mg/kg/day (calculated using *Tripterygium wilfordii Hook. F* chloroform extract). The 10 mg/kg/day dose for rats was equivalent to the clinical maximum dose of 1.5 mg/kg/day for humans recommended by the current Chinese National Drug Standard of TGT, WS3–B–3350–98–2011 (Chinese Pharmacopoeia Commission, 2011), which was calculated by the body surface area dosage conversion factors (Nair et al., 2018) and in consistence with the other reports (Shan et al., 2016; Gao et al., 2019). The 30 and 90 mg/kg doses were selected based on a literature (Gao et al., 2019).

Since the administration volume was set at 10 ml/kg/day, the TGT suspensions of 1.0, 3.0, and 9.0 mg extract per mL were freshly prepared by mixing an accurately weighed amount of the tablets powder with 0.5% CMC–Na solution through sonication for 30 min. The suspensions were efficiently vortex–mixed to ensure uniform dispersion before oral gavage administration.

### 2.5 Efficacy and Toxicity of TGT

#### 2.5.1 Study Design

**FIGURE 2:**
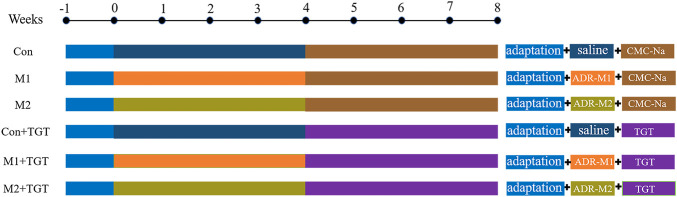
The experimental scheme for evaluating the efficacy and hepatotoxicity of TGT in adriamycin-induced-NS rats.

Successful induction of adriamycin–induced NS in rats (M1 and M2) was confirmed based on typical NS symptoms of oliguria, massive proteinuria, hypoalbuminemia, and hyperlipidemia (Supplementary Figure S1). The rats were then divided into six groups with seven rats each: three reference groups (Con, M1, M2) and three TGT treatment groups (Con + TGT, M1+TGT, and M2+TGT). The rats in the TGT treatment groups were administered TGT (10 mg/kg) by oral gavage once daily in the morning for 4 weeks, whereas the other rats received only the vehicle (0.5% CMC–Na solution) (Figure 2). Twenty–4 hours after the last dose (8 weeks after the first adriamycin injection), approximately 100 μl of blood was collected from all rats, followed by the immediate collection of 24 h urine samples for the determination of serum biochemical indices and urinary protein, respectively. After 24 h urine collection, each rat in the three TGT treatment groups received a single TGT oral gavage dose of 10 mg/kg and was sacrificed 1 h after administration. The kidney and liver samples from all groups were immediately harvested for histopathological examination and determination of the six key TGT components.

#### 2.5.2 Biochemical and Histopathological Analysis

Twenty–four–hour urinary protein (proteinuria) and serum biochemical indices (ALB, TP, TG, and TC) were determined using the corresponding kits according to the manufacturer’s instructions, on an Envision multimode microplate monitoring system. Kidney and liver tissue samples were fixed in 10% neutral buffered formalin, dehydrated in graded alcohol, embedded in paraffin, and sliced. The sliced 4 μm sections were subjected to routine hematoxylin and eosin (HE) staining and observed under a light microscope (Leica, Germany) for histopathological evaluation.

### 2.6 Pharmacokinetic Study

New sets of normal controls and two types of model rats in the three TGT treatment groups (*n* = 7) were included in the pharmacokinetic study of the six key TGT components. Before the experiment, all rats were fasted overnight with free access to water only. Each rat then received a single TGT oral gavage dose of 10 mg/kg. To determine the correlation between the contents of six TGT components in TGT and *in vivo* exposure, an additional 14 normal control rats were divided into two groups (*n* = 7) and given a single TGT dose of 30 or 90 mg/kg. Approximately 150 μl of blood was collected from the orbital venous plexus into heparinized tubes at 0, 0.083, 0.167, 0.25, 0.5, 0.75, 1, 2, 4, 6, 8, 10, 24, 34, and 48 h after administration. The technical personnel were well–trained in performing orbital venous plexus blood draws using a technique that imparts minimal pain to animals. All rats were intragastrically administered 1 ml of water after collection time points of 0.25, 0.5, 2, 4, and 6 h, and were allowed free access to water for fluid replacement throughout the experiment. The plasma was harvested by centrifuging the blood sample at 1,100 ×*g* for 10 min at 4°C, and then stored at –80°C until analysis.

A 70 μL aliquot of plasma was spiked with 10 μL of IS working solution (200 ng/ml in methanol) and 1 ml of acetonitrile solution. The mixture was then vortexed for 5 min and centrifuged at 16,000 ×*g* at 4°C for 10 min to extract the analytes and precipitate the endogenous protein materials. Next, 900 μL of the supernatant was collected and evaporated to dryness at 40 °C using a ZLS–1 vacuum centrifugal concentrator. The residue was reconstituted with 100 μl of methanol–water solution (60:40, v/v), and a 20 μl aliquot of the resulting supernatant after centrifugation was injected into the UHPLC–MS/MS system for analysis.

### 2.7 Tissue Distribution Study

For the tissue distribution study, new sets of normal controls and two types of model rats were assigned to the three TGT treatment groups (*n* = 7). Each rat was administered a single TGT oral gavage dose of 10 mg/kg after an overnight fast. Rats were sacrificed while under urethane anesthesia, by bleeding from the abdominal aorta 1 h after oral administration of TGT. Subsequently, the tissues (heart, liver, spleen, lung, kidney, and brain) were harvested, rinsed with ice–cold saline, blotted with filter paper, and weighed. Tissue samples were homogenized in ice–cold saline at a weight/volume (w/v) ratio of 1:9 using a JXFSTPRP–24 homogenizer (Shanghai, China).

To improve the recovery of the six key TGT components in tissues, liquid–liquid extraction was used for the pretreatment of the tissue samples as follows: a 70 µl aliquot of tissue homogenate was mixed with 10 μl of IS solution (200 ng/ml in methanol) and extracted with 1 ml of ethyl acetate solution by vortex–mixing for 5 min. A procedure similar to that used for plasma samples was then used to prepare the tissue samples for UHPLC–MS/MS analysis.

### 2.8 Excretion Studies

For urine and feces excretion studies, new sets of normal controls and two types of model rats assigned to the three TGT treatment groups (*n* = 7) were housed individually in metabolic cages, and each rat received a single TGT oral gavage dose of 10 mg/kg. Urine and fecal samples were collected at time windows of 0–6, 6–12, 12–24, 24–48, and 48–72 h after administration. The urine volume was measured and recorded, and the fecal samples were dried at 40°C, weighed accurately, pulverized into powder, and then homogenized in ice–cold saline (1:9, w/v).

For the bile excretion study, new sets of normal controls and two types of model rats assigned to the three TGT treatment groups (*n* = 7) were anesthetized by intraperitoneal injection of urethane solution. An abdominal incision was made, and a cannula was implanted into the bile duct. The incision was then closed by suturing. Blank bile samples were collected within 1 h before drug administration, and the drug–containing bile samples were harvested at time segments of 0–1, 1–2, 2–4, 4–6, 6–9, 9–24, and 24–33 h after a single TGT oral gavage dose of 10 mg/kg. Urine, fecal, and bile samples were prepared using a similar sample preparation procedure as tissue samples.

The selectivity, linearity, accuracy, precision, recovery, matrix effects, and stability of the developed method for the quantification of the six key TGT components in rat bio–samples were validated according to the FDA guidelines for bioanalytical method validation (Food and Drug Administration, 2018). The results of the method validation are summarized in Supplementary Figure S2–Supplementary Figure S6 and Supplementary Table S1–Supplementary Table S15.

### 2.9 Canonical Correlation Analysis of Pathology–Pharmacokinetics

CCA is a multivariate statistical model that identifies statistical dependencies between pairs of multivariate data. Two sets of variables can be converted into multiple pairs of canonical variates using the CCA. Among the canonical variate pairs, the first pair has the highest correlation (Koide-Majima and Majima, 2021). In this study, CCA was conducted to analyze the overall correlation between the severity of NS (proteinuria, ALB, TP, TC, and TG) and the PK characteristics (C _max_, AUC_0–t_, C _kidney_ or C _liver_) of the six key TGT components. Before the data mining application, the original NS data were normalized using min-max normalization to enable comparisons of these data by different units of measures. When the correlation significance level was less than 0.05, we considered the response data significant (Yu et al., 2017). In addition, the canonical loadings, which represent the correlation between an original variable and its canonical variable, contribute to determining the critical factors in each group of variables. As a rule, canonical loading with an absolute value greater than 0.3 was used to select the original variables that were thought to have a meaningful interpretation of the related canonical variables (Lambert and Durand, 1975).

### 2.10 Evaluation of Immunosuppressive Activity *in Vitro*

The mouse macrophage cell line RAW 264.7 (obtained from the Cell Bank of Chinese Academy of Sciences, Shanghai, China) was cultured in DMEM medium (Gibco, United States ) supplemented with 10% fetal bovine serum (FBS, Biological Industries, Israel) and 1% penicillin–streptomycin (P/S, Biological Industries, Israel) at 37°C in a humidified incubator with 5% CO_2_.

The inhibitory effects of the six target compounds (TPL, WA, WFG, WFT, WFD, and WFR) on the proliferation of LPS–stimulated RAW264.7 cells were examined by CCK8 assay. RAW 264.7 cells were seeded in 96–well plates (2×10^4^ cells/well) for 12 h, and then co–cultured with LPS (1 μg/ml) (Dai et al., 2021) and the six target compounds at different concentrations for 24 h. The medium was then removed and 10% CCK–8 working solution was added to each well, followed by incubation for 1 h. The optical density (OD) value of each well was measured at 450 nm using a microplate reader (Thermo Fisher, United States ). Cell viability was calculated as follows: cell viability (%) = (OD _sample_—OD _blank_)/(OD _control_—OD _blank_) × 100.

### 2.11 Evaluation of Hepatotoxicity *in Vitro*

The normal human liver cell line L02 (purchased from the Cell Bank of Chinese Academy of Sciences, Shanghai, China) was cultured in RPMI 1640 medium (Gibco, United States) containing 10% FBS and 1% P/S in a 5% CO_2_ incubator at 37°C.

To evaluate the hepatotoxicity, the effects of the four target compounds (TPL, WFG, WFT, and WFD) on L02 cell viability were determined using a CCK8 assay. Briefly, cells were cultured overnight in a 96–well plate (6×10^3^ cells/well), after which they were treated with TPL, WFG, WFT, and WFR in a series of concentrations for 48 h. Cell viability was determined according to the procedure described in Section 2.10.

### 2.12 Statistical Analysis

WinNonlin (version 7.0, Pharsight, St. Louis, MO, United States ) was used to analyze pharmacokinetic parameters. CCA was performed using SPSS Statistics 24.0 (IBM, United States ). Statistical differences between two groups were determined using GraphPad Prism 8.0 (GraphPad Software, Inc., San Diego, CA) via a two–tailed Student’s *t*–test. *p* < 0.05 was considered as statistical significance.

## 3 Results

### 3.1 Therapeutic Efficacy of the Multiple–Dose TGT Administration on Adriamycin–Induced–NS Rats

#### 3.1.1 Physical Parameters

**FIGURE 3:**
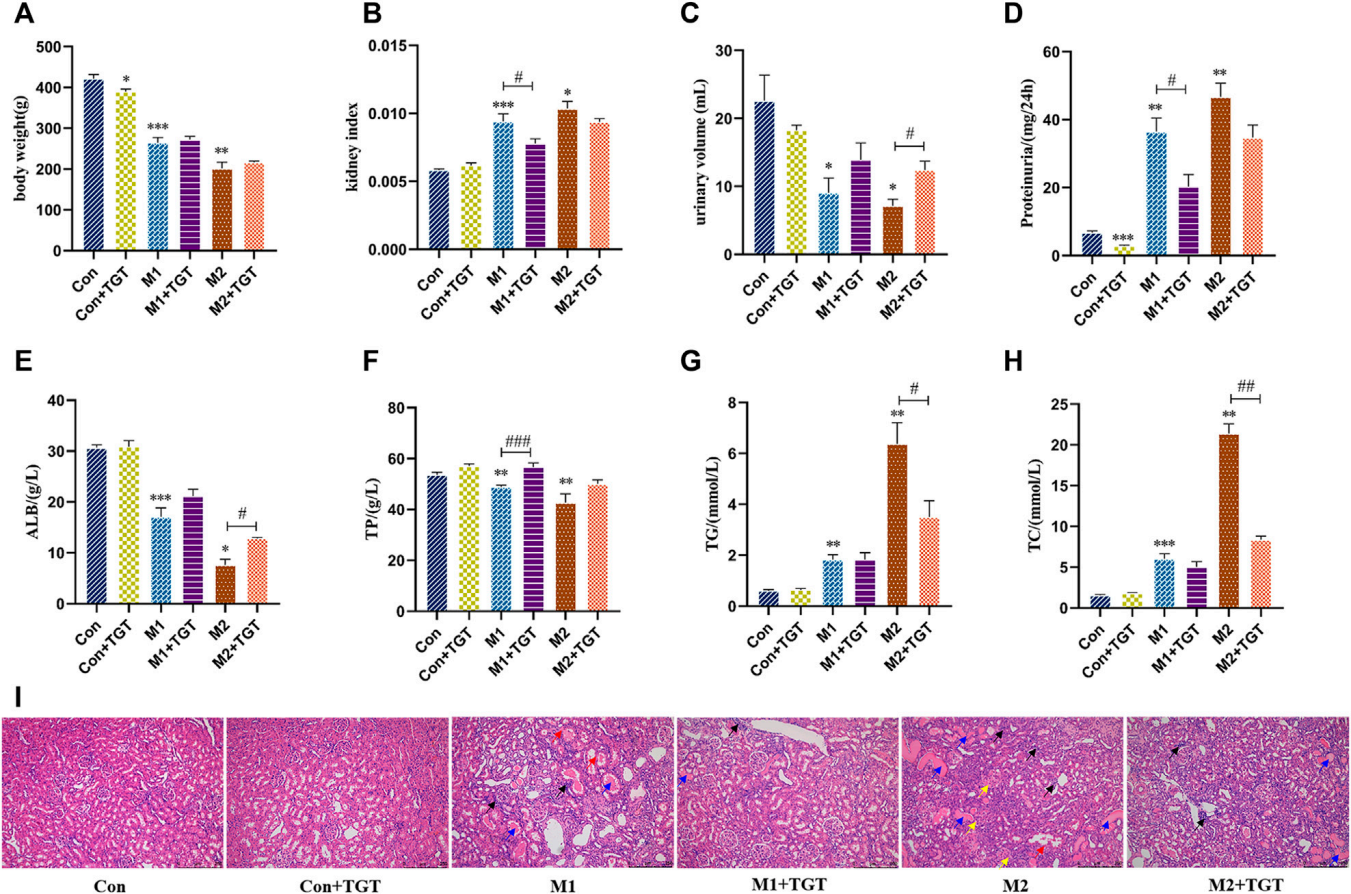
TGT ameliorated the symptoms of NS and renal pathological injuries in adriamycin-induced-NS rats after TGT oral gavage dose of 10 mg/kg/day for 4 weeks (means ± SD, *n* = 7). The body weight **(A)**, the kidney index **(B)**, the urine volume **(C)**, the 24-h proteinuria **(D)**, the serum ALB **(E)**, the serum TP **(F)**, the serum TG **(G)**, and the serum TC **(H)**, * *p* < 0.05, ** *p* < 0.01, *** *p* < 0.005 versus Con, ^**#**^ *p* < 0.05, ^**##**^ *p* < 0.01, ^**###**^ *p* < 0.005 versus M1 or M2. HE staining of the same field of section sample in kidney tissues **(I)**, interstitial inflammatory infiltrate (black arrow ↗), protein casts (blue arrow 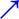), degeneration of renal tubular epithelial cells (red arrow 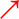), contraction of Bowman’s capsule (yellow arrow 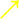) (magnification: ×100).

As shown in Figures 3A,B, the NS model rats (M1 and M2 groups) showed significant weight loss and kidney hyperplasia compared to the normal control rats (Con group). After treatment with TGT for 4 weeks, the weight loss and kidney hyperplasia in the M1+TGT and M2+TGT groups were decreased in comparison to those in the corresponding model control groups.

#### 3.1.2 Biochemical Analysis

Oliguria and proteinuria are diagnostic markers of glomerular filtration barrier dysfunction (Meyrier and Niaudet, 2018). In comparison with the corresponding control group, as shown in Figures 3C,D, the urine volumes of the NS model control rats (M1 and M2 groups) decreased significantly, while the 24 h urine protein excretion increased significantly. After treatment with TGT for 4 weeks, a significant increase in urine volume in the M2+TGT group and a remarkable decrease in urine protein excretion in the M1+TGT group were observed, indicating that TGT relieved the symptoms of oliguria and proteinuria in NS rats.

Hypoproteinemia and hyperlipidemia are also major clinical symptoms of NS (Shin et al., 2018). As shown in Figures 3E–H, the levels of serum ALB and TP declined significantly in the M1 and M2 group, whereas the serum TG and TC levels were significantly elevated. After treatment with TGT for 4 weeks, the ALB level in the M2+TGT group and the TP level in the M1+TGT group increased significantly, whereas the levels of TG and TC in the M2+TGT group decreased significantly, suggesting that TGT possessed therapeutic efficacy against hypoalbuminemia and hyperlipidemia in NS rats.

#### 3.1.3 HE Staining

As shown in Figure 3I, there was pronounced kidney pathology characterized by interstitial inflammatory infiltrates, protein casts, degeneration of renal tubular epithelial cells, and contraction of Bowman’s capsule. These changes were observed in the NS model rats (M1 and M2 groups), particularly in group M2. After treatment with TGT for 4 weeks, the pathological damage in the M1+TGT and M2+TGT groups was lower than that in the M1 and M2 groups.

### 3.2 Hepatotoxicity of TGT in Normal and Adriamycin–Induced–NS Rats After the Multiple–Dose TGT Administration

**FIGURE 4:**
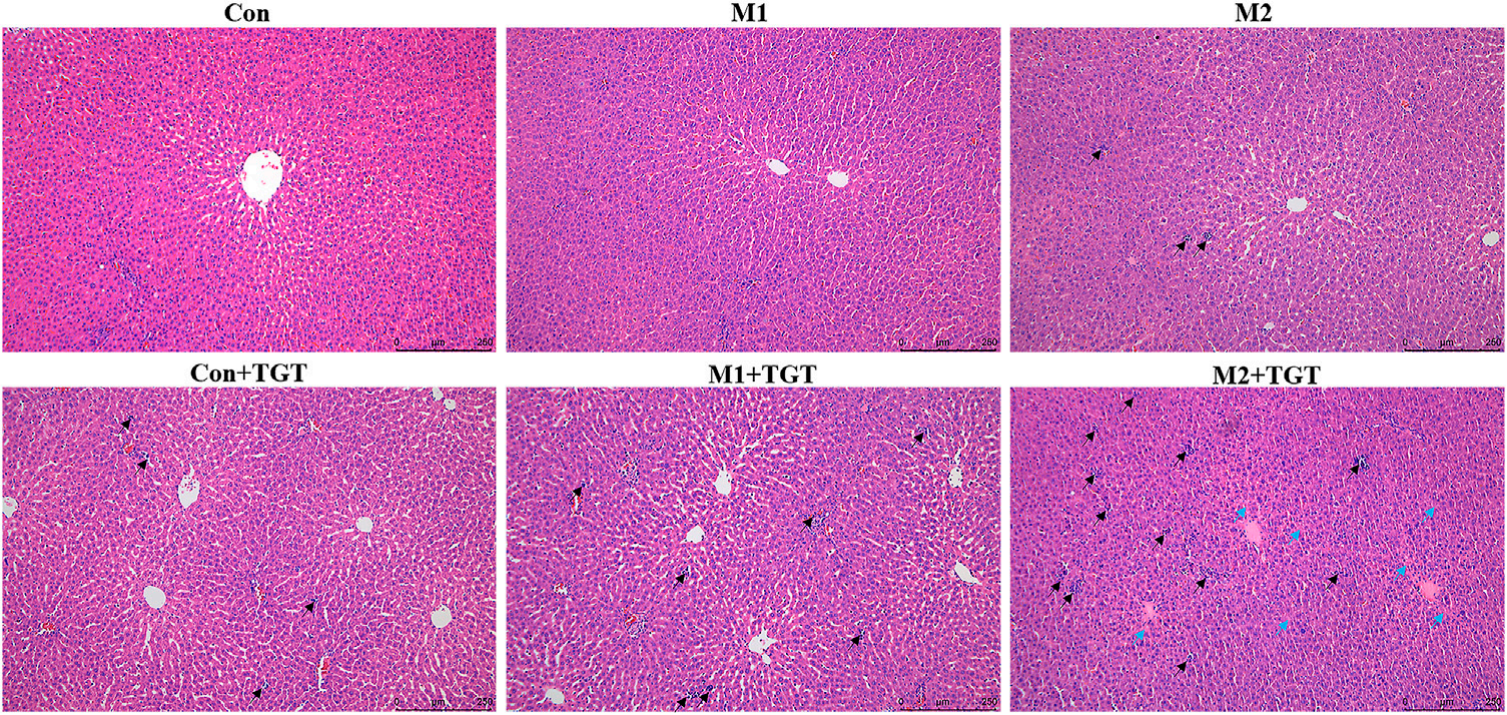
Representative pictures of HE staining of the liver tissues in the normal and two types of adriamycin-induced-NS rats assigned to three reference (Con, M1, M2), and three TGT treatment (Con + TGT, M1+TGT, and M2+TGT) groups after TGT oral gavage dose of 10 mg/kg/day for 4 weeks, inflammatory cell infiltration (black arrow **↗**), degeneration of hepatocytes (blue arrow 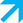) (magnification: ×100).

The histopathological micrographs of the livers in the three reference (Con, M1, M2), and three TGT treatment (Con + TGT, M1+TGT, and M2+TGT) groups are shown in Figure 4. In the Con, M1, and M2 groups, all rats presented intact liver architecture with no significant pathology except for slight interstitial inflammatory cell infiltration in group M2. After treatment with TGT for 4 weeks, mild interstitial inflammatory cell infiltration was observed in the Con + TGT group, which became more prominent in the M1 + TGT group. In addition to extensive inflammatory cell infiltration, severe hepatocyte degeneration was observed in the M2 + TGT group. These results suggested that NS, particularly advanced–stage NS, aggravated the hepatotoxicity caused by TGT.

### 3.3 Comparative Pharmacokinetic Study After the Single–Dose TGT Administration

**FIGURE 5:**
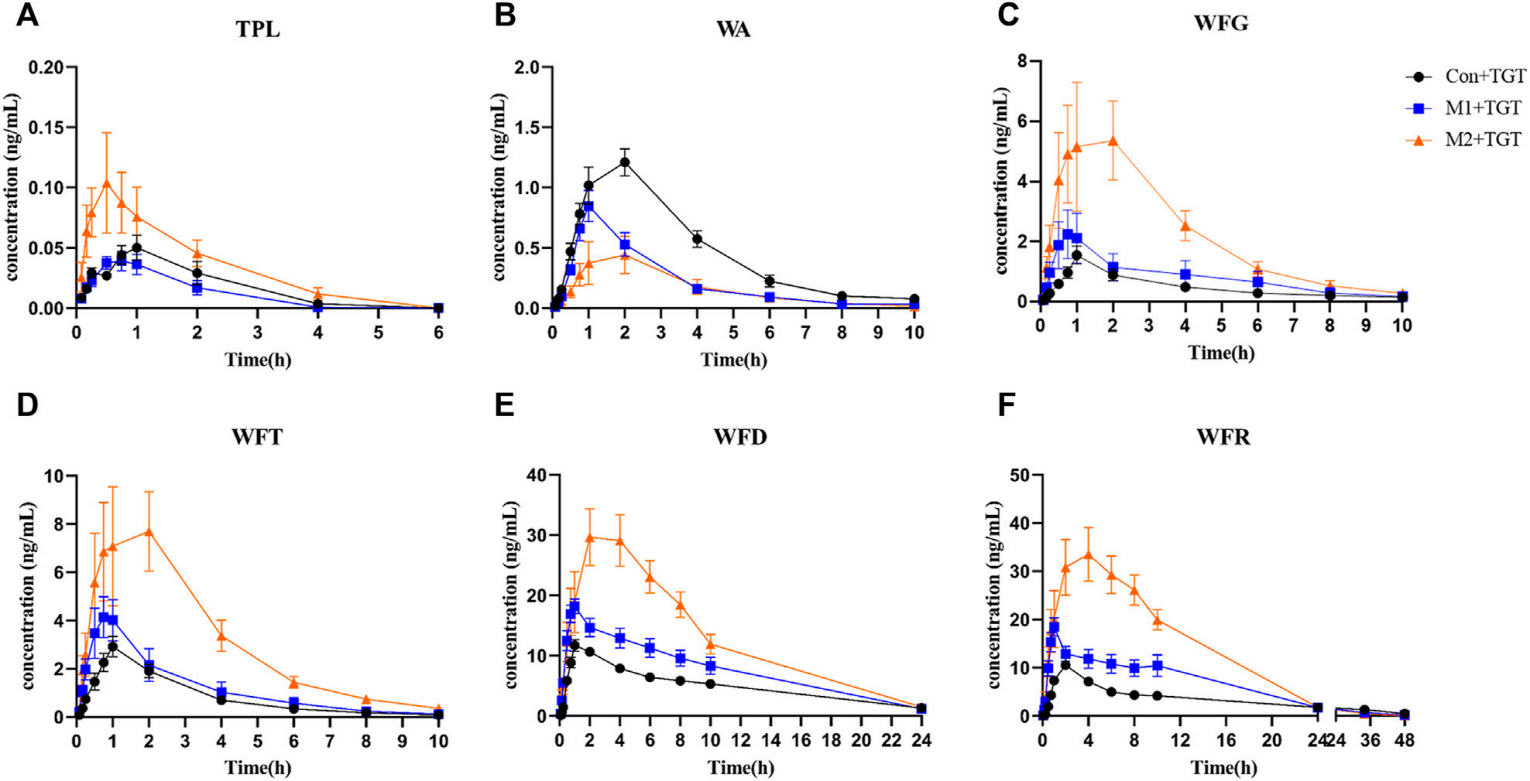
Plasma concentration–time profiles of the six key TGT components in the normal and two types of adriamycin-induced-NS rat after a single TGT oral gavage dose of 10 mg/kg (mean ± SD, n = 7). The normal rats, Con + TGT, black circle ●; the early-stage NS rats, M1+TGT, blue square 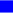; the advanced-stage NS rats, M2+TGT, orange triangle 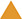. TPL **(A)**, WA **(B)**, WFG **(C)**, WFT **(D)**, WFD **(E)**, WFR **(F)**.

The mean plasma concentration–time curves and pharmacokinetic parameters of the six key TGT components (TPL, WA, WFG, WFT, WFD, and WFR) in normal and two types of NS rats assigned to the three TGT treatment groups (Con + TGT, M1+TGT, and M2+TGT) after a single TGT oral gavage dose of 10 mg/kg are shown in Figure 5 and Table 1, respectively. NS significantly altered the pharmacokinetic behavior of the six key TGT components. In the M1+TGT and M2+TGT groups, particularly in the M2+TGT group, higher plasma exposure of five key TGT components (TPL, WFG, WFT, WFD, and WFR) was found in the rats, characterized by higher C_max_, larger AUCs, smaller volume of distributions (Vd), and slower clearance rates (CL) than those in the normal rats (Con + TGT), whereas the WA showed the opposite trend.

**TABLE 1:** Pharmacokinetic parameters of the six key TGT components in the normal and two types of adriamycin-induced-NS rats after a single TGT oral gavage dose of 10 mg/kg (*n* = 7).

Components	Group	C_max _(ng/ml)	T_max _(h)	AUC_0-t_ (ng*h/mL)	AUC_0-∞_ (ng*h/mL)	*t*_1/2_ (h)	Vd (L/kg)	CL (L/h/Kg)	MRT_0-t _(h)	MRT_0-∞_ (h)
TPL	Con + TGT	0.05 ± 0.03	0.92 ± 0.13	0.10 ± 0.06	0.10 ± 0.03	0.73 ± 0.14	63.9 ± 28.6	59.1 ± 15.6	1.26 ± 0.31	1.46 ± 0.04
	M1 + TGT	0.05 ± 0.02	0.68 ± 0.19^**▲**^	0.07 ± 0.05	0.08 ± 0.06	0.87 ± 0.31	78.5 ± 43.2	61.8 ± 33.0	1.00 ± 0.25	1.43 ± 0.39
	M2 + TGT	0.12 ± 0.10	0.54 ± 0.29^**▲**^	0.19 ± 0.14^▲△^	0.29 ± 0.15	1.03 ± 0.85	46.1 ± 23.2	29.6 ± 26.2	1.31 ± 0.38	1.66 ± 1.17
WA	Con + TGT	1.30 ± 0.26	1.57 ± 0.53	4.67 ± 0.92	4.92 ± 1.01	2.11 ± 0.62	16.3 ± 6.13	5.35 ± 1.12	2.99 ± 0.48	3.48 ± 0.75
	M1 + TGT	0.87 ± 0.36^▲^	0.94 ± 0.12^▲^	2.19 ± 0.83^▲▲^	2.29 ± 0.93^▲▲^	1.70 ± 0.78	29.1 ± 12.1	12.9 ± 5.79^▲▲^	2.55 ± 0.39	2.91 ± 0.53
	M2 + TGT	0.50 ± 0.46^▲^	1.46 ± 0.60	1.62 ± 1.36^▲▲^	1.70 ± 1.32^▲▲^	1.29 ± 0.48^▲^	50.7 ± 54.2	27.2 ± 8.61^▲▲△△^	2.97 ± 0.49	3.09 ± 0.48
WFG	Con + TGT	1.59 ± 0.76	1.29 ± 0.49	4.86 ± 1.83	5.70 ± 2.26	3.53 ± 0.33	114 ± 15.6	24.4 ± 11.7	3.12 ± 0.29	4.88 ± 0.52
	M1 + TGT	2.42 ± 2.32	0.78 ± 0.16^▲▲^	6.33 ± 5.33	6.61 ± 5.52	2.68 ± 1.34^▲^	74.2 ± 40.4	21.3 ± 14.1	3.03 ± 0.52	3.88 ± 1.15^▲^
	M2 + TGT	6.75 ± 4.97^▲^	1.67 ± 0.58^△^	22.5 ± 12.3^▲△^	25.5 ± 11.9^▲△^	1.55 ± 0.32^▲▲^	12.0 ± 3.75^▲▲△^	5.24 ± 1.17^▲▲^	3.21 ± 1.12	3.07 ± 0.85^▲▲^
WFT	Con + TGT	2.97 ± 1.06	0.96 ± 0.09	8.64 ± 3.48	8.75 ± 3.49	2.57 ± 1.11	39.7 ± 17.2	11.6 ± 5.50	2.83 ± 0.62	3.01 ± 0.59
	M1 + TGT	4.62 ± 2.76	0.75 ± 0.19^▲^	12.6 ± 11.3	12.7 ± 11.3	2.39 ± 0.70	34.0 ± 15.6	11.0 ± 6.26	2.60 ± 0.66	2.70 ± 0.64
	M2 + TGT	9.21 ± 5.76	1.86 ± 1.07^▲△△^	33.7 ± 15.2^▲▲△△^	33.8 ± 15.2^▲▲△△^	2.82 ± 0.56	14.8 ± 12.7^▲△^	3.36 ± 2.35^▲▲△△^	3.73 ± 1.52^△^	3.81 ± 1.60
WFD	Con + TGT	12.5 ± 2.01	1.43 ± 0.53	137 ± 24.8	138 ± 25.4	8.24 ± 1.36	10.6 ± 1.49	0.91 ± 0.17	10.9 ± 2.01	11.2 ± 2.29
	M1 + TGT	19.0 ± 3.66^▲▲^	1.41 ± 1.13	197 ± 68.1^▲^	197 ± 68.2^▲^	7.29 ± 1.22	6.94 ± 1.78^▲▲^	0.68 ± 0.20^▲^	8.57 ± 1.01^▲^	8.72 ± 1.05^▲▲^
	M2 + TGT	32.7 ± 11.3^▲▲▲△△^	3.71 ± 2.43^▲△△^	328 ± 53.5^▲▲▲△△^	329 ± 53.2^▲▲▲△△^	8.63 ± 4.77	4.79 ± 3.04^▲▲^	0.38 ± 0.06^▲▲▲△△^	8.13 ± 2.02^▲^	8.29 ± 2.06^▲^
WFR	Con + TGT	10.6 ± 1.52	1.86 ± 0.38	137 ± 20.5	141 ± 21.6	14.4 ± 1.93	26.3 ± 5.96	1.26 ± 0.19	16.1 ± 1.78	18.4 ± 2.18
	M1 + TGT	19.4 ± 5.19^▲▲^	1.57 ± 1.13	220 ± 92.6^▲^	221 ± 92.3^▲^	8.46 ± 1.35^▲▲▲^	11.4 ± 5.17^▲▲▲^	0.90 ± 0.30^▲^	10.6 ± 1.06^▲▲▲^	10.9 ± 1.27^▲▲▲^
	M2 + TGT	37.4 ± 13.6^▲▲▲△△^	4.33 ± 2.34^▲△^	433 ± 86.4^▲▲▲△△^	433 ± 86.5^▲▲▲△△^	6.38 ± 1.97^▲▲▲△^	3.83 ± 1.38^▲▲▲△△^	0.42 ± 0.08^▲▲▲△△^	8.33 ± 2.09^▲▲▲△^	8.37 ± 2.09^▲▲▲△^
The normal and two types of adriamycin-induced-NS rats are assigned to Con + TGT, M1+TGT, and M2+TGT groups, respectively. ^▲^ *p* < 0.05, ^▲▲^ *p* < 0.01, ^▲▲▲^ *p* < 0.005 vs Con + TGT, ^△^ *p* < 0.05, ^△△^ *p* < 0.01 vs M1+TGT.											

The plasma concentration–time profiles and corresponding pharmacokinetic parameters of the six TGT components in normal rats after a single oral gavage dose of 10, 30, or 90 mg/kg TGT are illustrated in Supplementary Figure S7 and Supplementary Table S16, respectively. The *in vivo* exposure of the six key TGT components showed a dose–dependent trend and increased correspondingly with an increase in dose.

### 3.4 Comparative Tissue Distribution Study After the Single–Dose or Multiple–Dose TGT Administration

**FIGURE 6:**
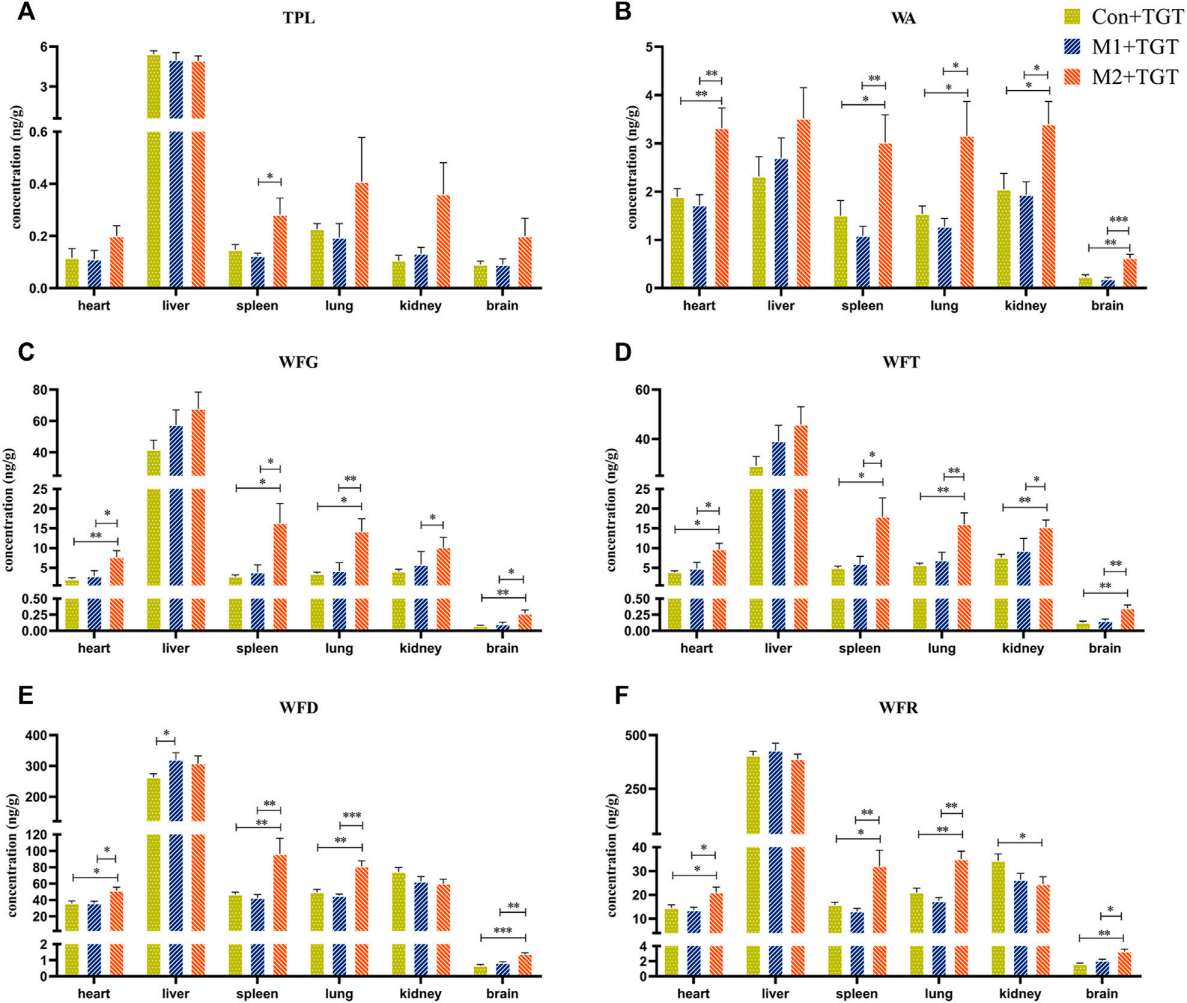
Tissue distributions of the six key TGT components in the normal and two types of adriamycin-induced-NS rats 1 hour after a single TGT oral gavage dose of 10 mg/kg (mean ± SD, *n* = 7). Con + TGT, the normal rats; M1 + TGT, the early-stage NS rats; M2 + TGT, the advanced-stage NS rats. TPL **(A)**, WA **(B)**, WFG **(C)**, WFT **(D)**, WFD **(E)**, WFR **(F)**, ∗*p* < 0.05, ∗∗*p* < 0.01, ∗∗∗*p* < 0.005 versus Con + TGT or M1+TGT.

The tissue contents of the six key TGT components (TPL, WA, WFG, WFT, WFD, and WFR) in the normal and two types of NS rats assigned to the three TGT treatment groups (Con + TGT, M1+TGT, and M2+TGT) 1 h after a single TGT oral gavage dose of 10 mg/kg are shown in Figure 6. The contents of these six components were highest in the liver, followed by the kidney, spleen, lung, heart, and brain. The levels of WA, WFG, WFT, and WFD in the liver were elevated in the M1+TGT and M2+TGT groups, especially in the M2+TGT group. The renal contents of WA, WFG, and WFT in the M2+TGT group were significantly higher than those in the Con + TGT and M1+TGT groups, whereas the WFR in the M2+TGT group was significantly lower than that in the Con + TGT group.

The WA, WFG, WFT, WFD, and WFR contents in the heart, spleen, lung, and brain were significantly higher in the M2+TGT group than those in the other two treatment groups. In addition, a significantly elevated TPL content in the spleen was observed in the M2+TGT group compared to that in the M1+TGT group.

Collectively, these data showed that the tissue contents of the six key TGT components in the heart, liver, spleen, lung, kidney, and brain increased in NS rats, particularly in advanced–stage NS rats after single–dose TGT administration.

**FIGURE 7:**
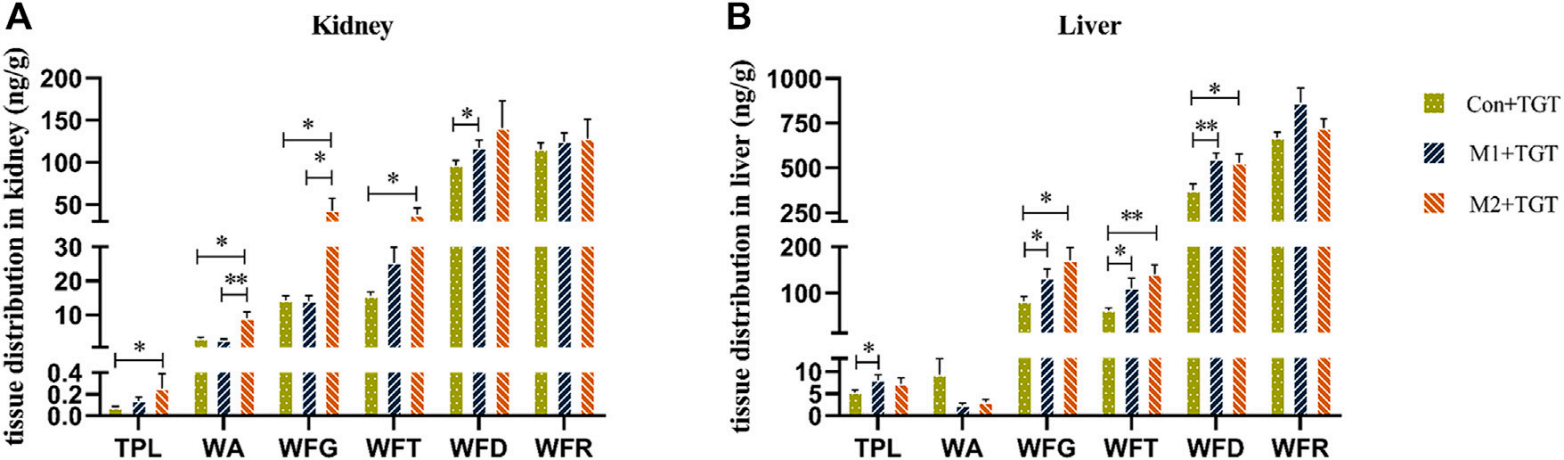
Tissue distributions of the six key TGT components in the normal and two types of adriamycin-induced-NS rats 1 hour following the last dose after TGT oral gavage dose of 10 mg/kg/day for 4 weeks (means ± SD, *n* = 7). Con + TGT, the normal rats; M1 + TGT, the early-stage NS rats; M2 + TGT, the advanced-stage NS rats. Kidney **(A)**, Liver **(B)**, ∗*p* < 0.05, ∗∗*p* < 0.01 versus Con + TGT or M1+TGT.

Figure 7 shows the cumulative exposure of the six key TGT components in the kidney and liver in the three TGT treatment (Con + TGT, M1+TGT, and M2+TGT) groups after once daily oral gavage dosing with 10 mg/kg TGT for 4 weeks. Compared with the Con + TGT group, the cumulative exposure of TPL, WA, WFG, WFT, and WFD in the kidney, and TPL, WFG, WFT, and WFD in the liver increased significantly in the M1+TGT and M2+TGT groups, particularly in the M2+TGT group. The increased exposure of these key TGT components in the kidney and liver with the aggravation of NS status likely contributed to the anti–NS effects as well as the obvious hepatotoxicity described in Section 3.2.

### 3.5 Comparative Excretion Studies After the Single–Dose TGT Administration

**FIGURE 8:**
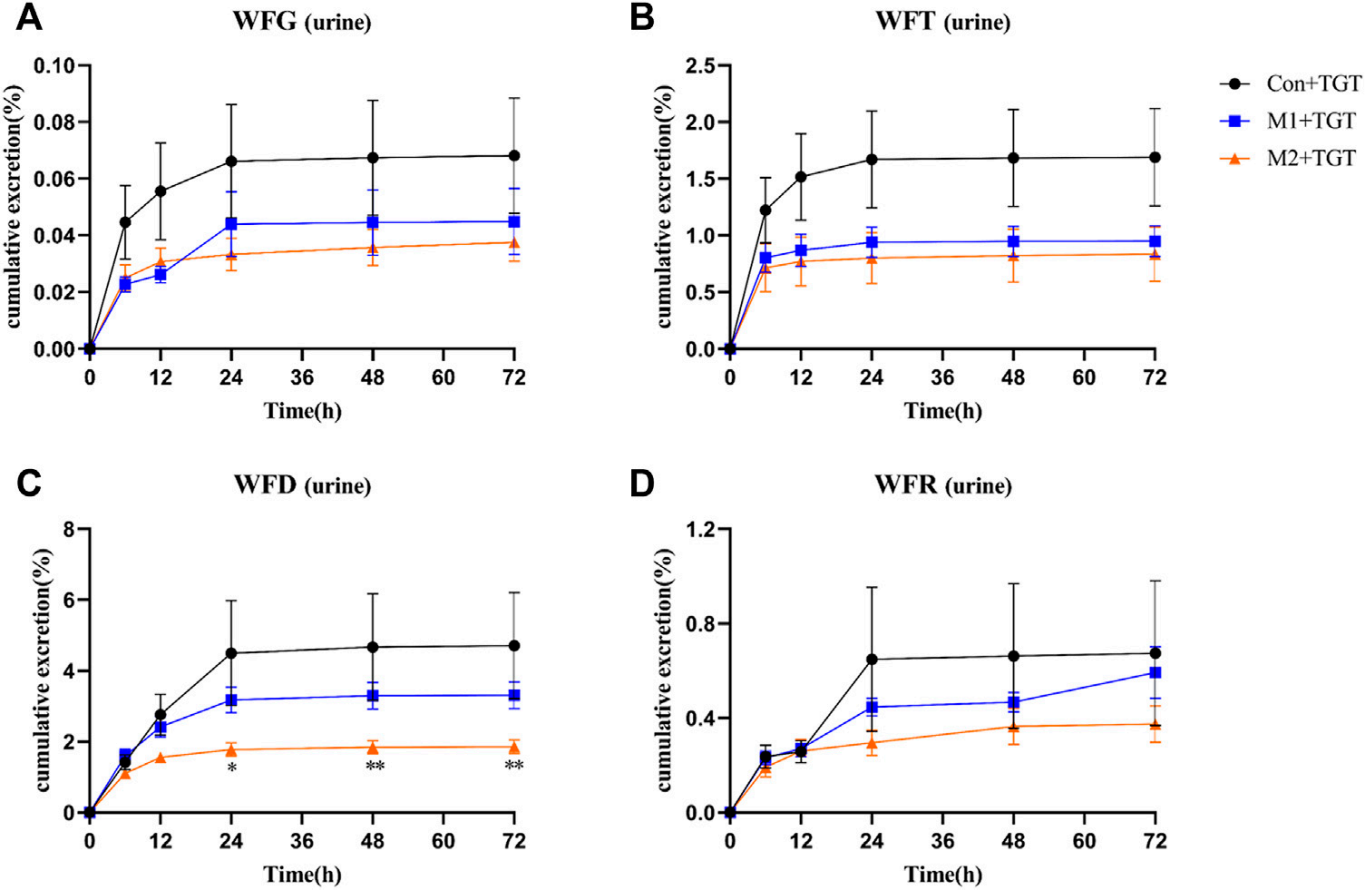
The cumulative urine excretion ratios of the four key TGT components in the normal and two types of adriamycin-induced-NS rats after a single TGT oral gavage dose of 10 mg/kg (mean ± SD, *n* = 7). The normal rats, Con + TGT, black circle ●; the early-stage NS rats, M1+TGT, blue square 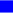; the advanced-stage NS rats, M2+TGT, orange triangle 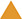. WFG **(A)**, WFT **(B)**, WFD **(C)**, WFR **(D)**, ∗*p* < 0.05, ∗∗*p* < 0.01 versus Con + TGT.

**FIGURE 9:**
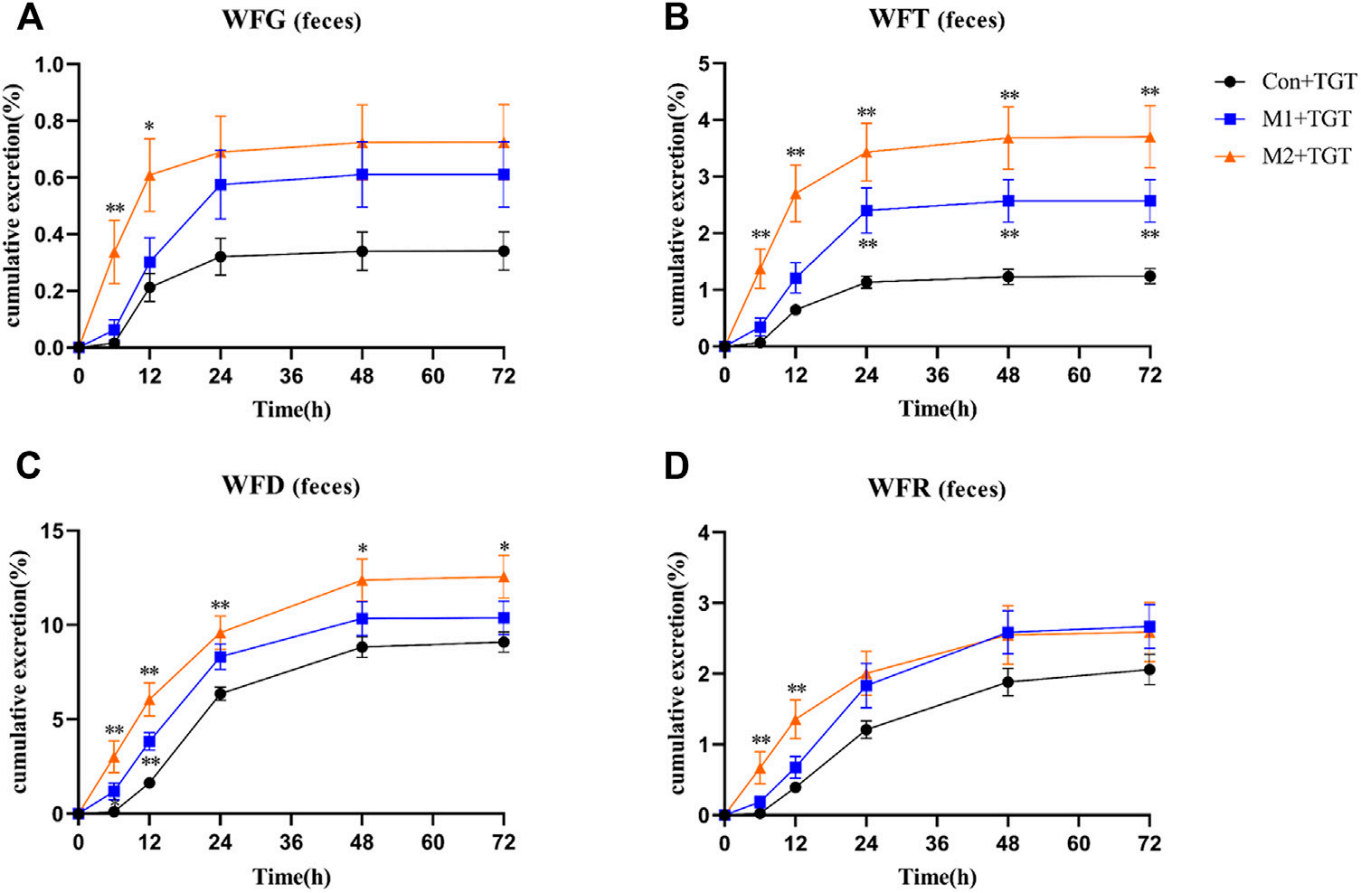
The cumulative feces excretion ratios of the four key TGT components in the normal and two types of adriamycin-induced-NS rats after a single TGT oral gavage dose of 10 mg/kg (mean ± SD, *n* = 7). The normal rats, Con + TGT, black circle ●; the early-stage NS rats, M1+TGT, blue square 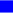; the advanced-stage NS rats, M2+TGT, orange triangle 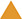. WFG **(A)**, WFT **(B)**, WFD **(C)**, WFR **(D)**, ∗*p* < 0.05, ∗∗*p* < 0.01 versus Con + TGT.

**FIGURE 10:**
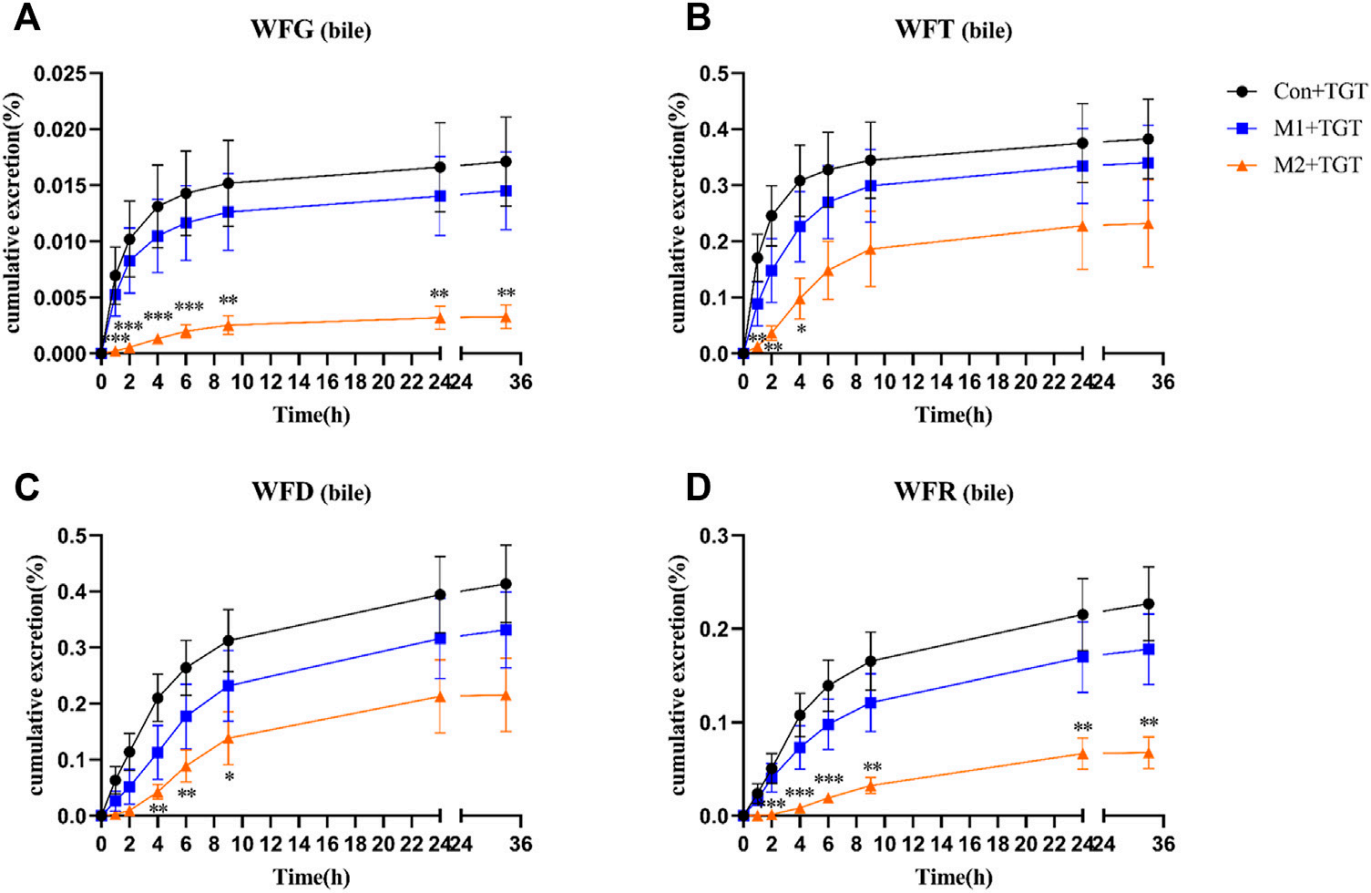
The cumulative bile excretion ratios of the four key TGT components in the normal and two types of adriamycin-induced-NS rats after a single TGT oral gavage dose of 10 mg/kg (mean ± SD, *n* = 7). The normal rats, Con + TGT, black circle ●; the early-stage NS rats, M1+TGT, blue square 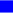; the advanced-stage NS rats, M2+TGT, orange triangle 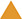. WFG **(A)**, WFT **(B)**, WFD **(C)**, WFR **(D)**, ∗*p* < 0.05, ∗∗*p* < 0.01, ∗∗∗*p* < 0.005 versus Con + TGT.

The urine, feces, and bile excretion profiles of six key TGT components in normal and two types of NS rats assigned to the three TGT treatment groups (Con + TGT, M1+TGT, and M2+TGT) after a single TGT oral gavage dose of 10 mg/kg are illustrated in Figures 8–10. When the normal and NS rats were administered the TGT, only four key TGT components (WFG, WFT, WFD, and WFR) of six tested were detected in the urine, feces, and bile samples. By contrast, TPL and WA were not detected or were below the limit of quantitation in all three bio–samples throughout the validated concentration ranges. Therefore, metabolism is a major method of excretion for these components.

As shown in Figure 8, only a small amount of the detected TGT components (WFG, WFT, WFD, and WFR) were excreted in their original form through urine. The 72 h cumulative urine excretion ratios of the detected TGT components further decreased with the increased severity of NS in rats.

The amounts of the detected TGT components (WFG, WFT, WFD, and WFR) excreted in their original form through feces were higher than those excreted in urine. However, the 72 h cumulative feces excretion ratios of the detected TGT components further increased with the increased severity of NS in rats (Figure 9).

Only a small portion of the detected TGT components (WFG, WFT, WFD, and WFR) were excreted into the bile in their original form. In addition, the 33 h cumulative bile excretion ratios of the detected TGT components also further decreased with the increased severity of NS in rats (Figure 10).

### 3.6 Canonical Correlation Analysis Between the Severity of NS and *in Vivo* Exposure of Six Key TGT Key Components

The correlations of the first canonical variate pair between NS and C_max_, NS and AUC_0–t_, NS and C_kidney_, and NS and C_liver_ were statistically significant (*F* = 3.105, *p* < 0.05; *F* = 2.825, *p* < 0.05; *F* = 3.859, *p* < 0.05; *F* = 2.201, *p* < 0.05, respectively). The first canonical correlation coefficients were 0.929, 0.933, 0.982, and 0.951, respectively, indicating that the four sets of first canonical variate pairs were positively correlated.

Subsequently, the original variables of canonical loadings with absolute values greater than 0.3 were selected as the key factors in each group of variables. As shown in Table 2, for NS and C_max_, five NS indicators (proteinuria, ALB, TP, TC, and TG) affected the peak exposure of the six TGT components in plasma (TPL, WA, WFG, WFT, WFD, WFR). For NS and AUC_0–t_, five NS indicators (proteinuria, ALB, TP, TC, and TG) collectively affected the total exposure of the five TGT components (WA, WFG, WFT, WFD, WFR) in plasma. For NS and C_kidney_, three NS indicators (proteinuria, ALB, and TG) affected the exposure of the six TGT components (TPL, WA, WFG, WFT, WFD, and WFR) in the kidney. For NS and C_liver_, five NS indicators (proteinuria, ALB, TP, TC, and TG) also affected the exposure of the four TGT components (TPL, WFG, WFT, and WFD) in the liver.

**TABLE 2:** Canonical structures of the first pair of canonical variables between the severity of NS and *in vivo* exposure of six key TGT key components (*n* = 7).

Group	*U*	Loading	*V*	Loading	Rc	F	P
NS and C_max_	Proteinuria	−0.678	TPL	−0.587	0.929	3.105	0.0003
	ALB	0.838	WA	0.632			
	TP	0.806	WFG	−0.584			
	TC	−0.913	WFT	−0.555			
	TG	−0.644	WFD	−0.692			
			WFR	−0.679			
NS and AUC_0-t_	Proteinuria	−0.844	TPL	−0.230	0.933	2.825	0.001
	ALB	0.896	WA	0.703			
	TP	0.931	WFG	−0.565			
	TC	−0.982	WFT	−0.674			
	TG	−0.796	WFD	−0.888			
			WFR	−0.914			
NS and C_kidney_	Proteinuria	−0.362	TPL	−0.898	0.982	3.859	0.00006
	ALB	0.341	WA	−0.789			
	TP	0.209	WFG	−0.974			
	TC	−0.242	WFT	−0.947			
	TG	−0.792	WFD	−0.921			
			WFR	−0.91			
NS and C_liver_	Proteinuria	−0.929	TPL	−0.390	0.951	2.201	0.014
	ALB	0.759	WA	−0.297			
	TP	0.842	WFG	−0.376			
	TC	−0.707	WFT	−0.432			
	TG	−0.722	WFD	−0.392			
			WFR	−0.268			

*p *< 0.05 means a significant difference.

### 3.7 Immunosuppressive Activities of the Six Target Compounds in RAW 264.7 Cells

The inhibitory effects of the six target compounds on the proliferation of LPS–stimulated RAW264.7 cells are shown in Supplementary Figure S8. The results showed that TPL (5–100 nM), WFT (50–2,500 nM), and WFD (0.25–2.5 μM) exhibited strong immune–suppressive activities (*p* < 0.05) in a dose–dependent manner. WA at a concentration of 5 μM, as well as WFG and WFR at a concentration of 25 μM significantly suppressed the proliferation of LPS–stimulated RAW264.7 cells.

### 3.8 Hepatotoxic Effects of the Four Target Compounds in L02 Cells

The half maximal inhibitory concentration (IC_50_) values of the four target compounds were finally calculated from the concentration–cell viability curves. As shown in Supplementary Figure S9, TPL had a low IC_50_ value of 26.8 nM in L02 cells, exhibiting extremely strong hepatotoxic potential. In addition, WFG, WFT, and WFD also showed potent hepatotoxicity with single–digit micromolar IC_50_ values of 2.6, 2.3, and 1.3 μM, respectively.

## 4 Discussion

TGT have been prescribed for patients with CKD for more than 4 decades with sufficient clinical efficacy (Shao et al., 2021). In this study, the efficacy of TGT on the early–and advanced–stage NS rats was further confirmed (Figure 3). However, when a drug with a narrow therapeutic window is used clinically, other adverse effects can appear. Liver injuries were observed in rats after oral administration of TGT for 4 weeks (Figure 4), which is consistent with a previous report (Zhu et al., 2013). In addition, our group found that liver damage caused by TGT was further aggravated by the progression of NS in rats (Figure 4).

The pharmacokinetic behavior of the key bioactive components in TCMs has been used to predict their efficacy and potential toxicity (Zhang et al., 2018; Gopi and Balakrishnan, 2021). Previous studies on TGT pharmacokinetics have mainly focused on healthy animals, or animals with rheumatoid arthritis. Of note, the severity of rheumatoid arthritis was positively correlated with plasma exposure to WFG and WFR in rats after TGT administration (Su et al., 2015a; Lin et al., 2021; Zhao et al., 2021). Thus, it is rational to investigate the influence of NS on the key TGT components, as NS is another clinical indication for TGT.

NS can cause a gradual loss of renal function and a reduction in glomerular filtration rate, which then decreases the renal clearance of drugs (Torres et al., 2021; Politano et al., 2020). This may explain the significant decrease in urinary excretion ratios of the four detected TGT components (WFG, WFT, WFD, and WFR) that we observed in NS rats (Figure 8). Kidney disease can change not only the renal elimination but also the non–renal disposition of drugs (Lalande et al., 2014). The metabolism of key TGT components is primarily mediated by hepatic cytochrome P450 (CYP450) enzymes (Li et al., 2015). For example, CYP3A4 is predominantly involved in the metabolism of TPL (Xu et al., 2018). Researchers have found that the gene expression of some CYP450 enzyme isoforms (CYP3A4, CYP1A2, CYP2C9, et al.) and the activity of some CYP450 enzyme isoforms (CYP3A subtypes) decrease in kidney disease (Dong et al., 2020; Ladda and Goralski, 2016), leading to a reduction in drug metabolism and bile excretion in their prototypic or metabolite form. Consistent with previous reports, we found that the bile excretion ratios of the four detected key TGT components (WFG, WFG, WFD, and WFR) in their original form were significantly reduced in NS rats (Figure 10). Additionally, the decrease in the expression of some kidney uptake transporters (Oat1 and Oct2) and liver uptake transporters (Oatp1, 2, and 4) in nephropathy rats (Sun et al., 2006) may also reduce drug clearance.

Reducing both renal and non–renal elimination of key TGT components likely changes the *in vivo* exposure of these components. The plasma exposure of five key TGT components (TPL, WFG, WFT, WFD, and WFR) and the tissue contents of six key TGT components (TPL, WA, WFG, WFT, WFD, and WFR) in the heart, liver, spleen, lung, kidney, and brain increased significantly after single–dose TGT administration in NS rats (Figures 5, 6 and Table 1), consistent with previous reports that the plasma exposure of certain drugs increased when kidney function declined (Albrecht et al., 2017; van der Aart-van der Beek et al., 2021). Furthermore, the cumulative exposure of four key TGT components (TPL, WFG, WFT, and WFD) in the kidney and liver also increased after multi–dose TGT administration in NS rats, particularly advanced–stage NS rats (Figure 7). Drug accumulation in the target tissues could enhance therapeutic efficacy, but also result in unexpected toxicity (Pellegatti and Pagliarusco, 2011), which may account for the production of anti–NS effects and the increased hepatotoxicity of TGT with the increased severity of NS.

In this study, the NS rat model was induced with adriamycin, a routine chemotherapy agent that commonly causes gastrointestinal injuries (Kaczmarek et al., 2012). Small intestinal damage characterized by a decrease in the length and number of villi in the jejunum and ileum was also observed in NS rats (Supplementary Figure S10), which may be responsible for the increase in fecal excretion of four detected key TGT components (WFG, WFT, WFD, and WFR) in NS rats (Figure 9). Although the absorption of these four TGT components was reduced in the NS state, elimination was slowed leading to high exposure *in vivo*. Excessive or long–term use of TGT for the treatment of NS may still pose hazardous risks.

CCA was used to screen the TGT active components related to their anti–NS and hepatotoxic effects in this study, and the immunosuppressive activity and hepatotoxicity were verified *in vitro*. TPL, WA, WFG, WFT, WFD, and WFR were identified as the material bases for the treatment of NS, while TPL, WFG, WFT, and WFD were recognized as hepatotoxic material bases (Table 2, Supplementary Figure S8, Supplementary Figure S9). These results would help to better understand the mechanisms of the action of TGT.

Due to the narrow therapeutic window of TGT, unexpected tissue toxicity, particularly hepatotoxicity, need to be considered when treating NS patients. In addition, therapeutic drug monitoring of key TGT components should be included in clinical practice to reduce the risk of adverse reactions. Finally, the pharmacokinetic study of TGT in the clinic should consider the pathological status for further credible guidance information on clinical medication.

## 5 Conclusion

In summary, TGT exhibited clear therapeutic efficacy in NS rats, but aggravated hepatotoxicity under the NS state. The potential mechanism was related to the significant increase of *in vivo* exposure of the six key TGT components in NS rats. TPL, WA, WFG, WFT, WFD, and WFR were identified as the anti–NS material bases of TGT, whereas TPL, WFG, WFT, and WFD were recognized as hepatotoxic material bases. This work will be helpful for the rational clinical applications of TGT.

## Data Availability

The original contributions presented in the study are included in the article/Supplementary Material, further inquiries can be directed to the corresponding authors.
