# Supplementary material for: Differences in Multicomponent Pharmacokinetics, Tissue Distribution, and Excretion of Tripterygium Glycosides Tablets in Normal and Adriamycin–Induced Nephrotic Syndrome Rat Models and Correlations With Efficacy and Hepatotoxicity
Source: Front Pharmacol. 2022 Jun 9;13:910923. doi: 10.3389/fphar.2022.910923 (PMC9221999; doi:10.3389/fphar.2022.910923)
Supplement: Supplementary file 1 [file DataSheet1.docx]

**Supplementary materials**

**1. Biochemical evaluation criteria of the adriamycin-induced-NS rats**

As shown in **Fig. S1**, the urine volumes, and the levels of serum ALB and TP in the model control rats (M1 and M2) were decreased significantly while the 24-h urine protein excretions, the levels of TG, and TC in serum were increased significantly compared with those in the normal control rats (Con).

**
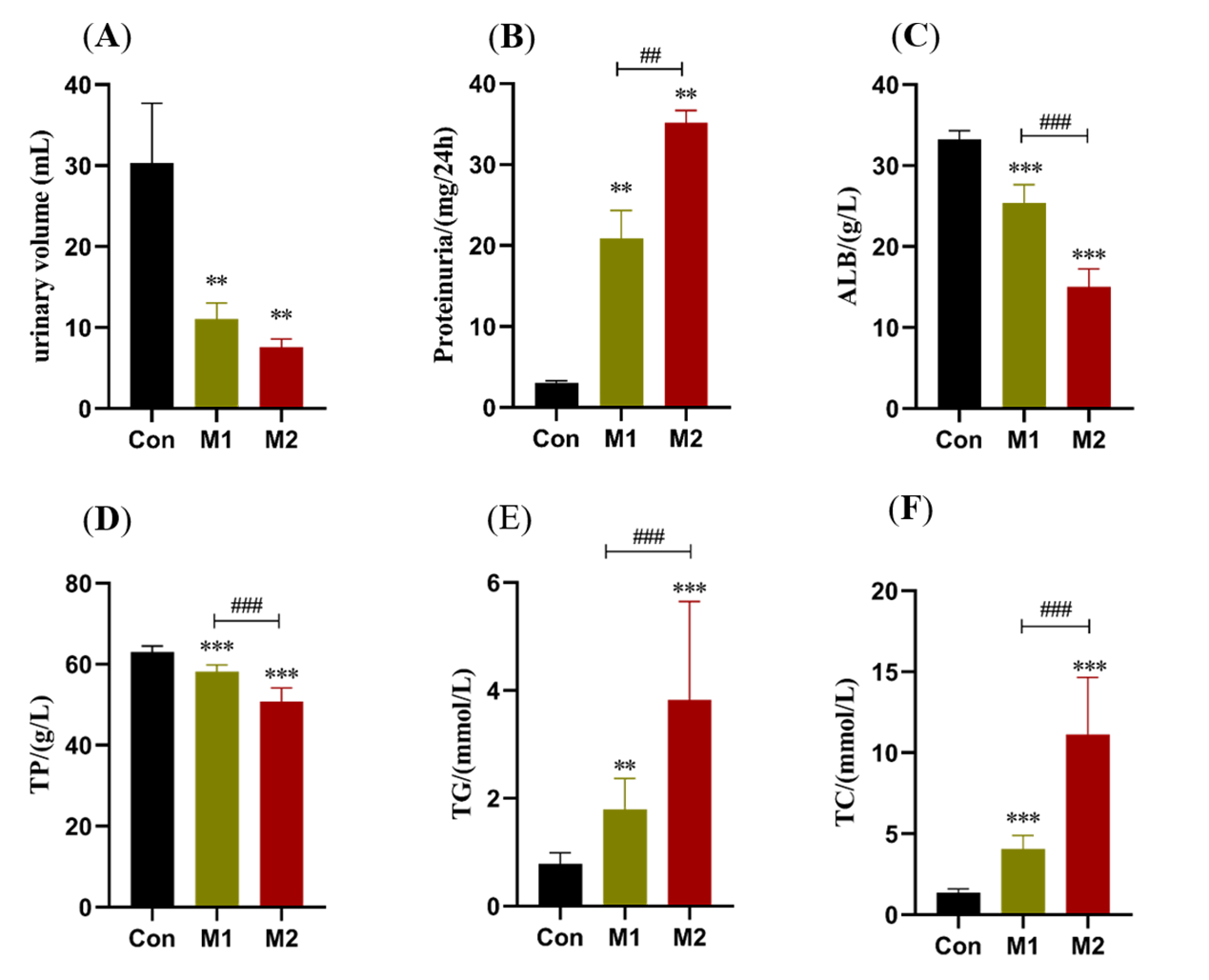
**

**Fig. S1** Biochemical evaluation indexes of two types of adriamycin-induced-NS rats at the end of the 4th week after the first adriamycin injection (mean ± SD, n = 7). The urine volume (**A**), the 24-hour proteinuria (**B**), the serum ALB (**C**), the serum TP (**D**), the serum TG (**E**), and the serum TC (**F**), *p < 0.05, **p < 0.01, ***p < 0.005 versus Con, ##p < 0. 01, ###p < 0.005 versus M1.

**2. Method validation**

**2.1. Preparation of standard solutions**

Standard stock solutions of TPL, WA, WFG, WFT, WFD, WFR and IS were prepared with methanol by dissolving the accurately weighed amounts in volumetric flasks to make a concentration of 1 mg/ml, respectively.

**2.2. Selectivity**

Selectivity was investigated by analyzing blank rat plasma / liver / urine / feces / bile samples from six individuals and comparing them with the corresponding spiked samples. Representative chromatograms of blank rat plasma / liver / urine / feces / bile samples, blank plasma / liver /urine /feces / bile samples spiked with TPL, WA, WFG, WFT, WFD, WFR and IS, and plasma / liver / urine / feces / bile samples after oral administration of TGT are shown in **Figs. S2-S6**. The results indicated no interfering peaks in the regions of the analytes and IS, and the method showed high selectivity.

**2.3. Linearity and LLOQ**

Calibration curves were determined by plotting the peak area ratio of each analyte to the IS (Y-axis) against the nominal value of analyte (X-axis) and evaluated for linearity using a weighted (1/x^2^) least-squares regression analysis. Lower limit of quantification (LLOQ) was defined as the lowest concentration of the calibration curve. The acceptance criterion for each back-calculated standard concentration is within 15% deviation from the nominal value and 20% for LLOQ, and the correlation coefficient (R^2)^ value of the curve should be greater than 0.9801. As summarized in **Tables S1−S5**, all calibration curves were linear over the tested concentration ranges. The correlation coefficient (R^2^) values of the curves were all greater than 0.9801, indicating that the analysis method showed good linearity. And the LLOQs for TPL, WA, WFG, WFT, WFD and WFR were sufficient for blood pharmacokinetics, tissue distribution, and excretion studies.

**2.4. Precision, accuracy, recovery and matrix effect**

Inter- and intra-day precision and accuracy for the assay were characterized by the performance of four levels of QC runs on three validation days. On each day, five replicates ere analyzed together with an independently prepared calibration curve. The precision was expressed as the relative standard deviation (RSD%) of measured concentrations. The accuracy was expressed as a percentage error of measured concentrations versus nominal concentrations (RE%).

The recovery was evaluated by comparing the peak area ratio of each analyte to the IS obtained from extracted spiked samples with those of the post-extracted spiked samples. The matrix effect was evaluated by comparing the peak areas of the post-extracted spiked QC samples with those of corresponding standard solutions. These procedures were repeated for five replicates at four QC concentration levels.

Precision, accuracy, recovery and matrix effect for the six analytes are summarized in **Tables S6-S10**. The data indicated that the precision and accuracy in plasma / liver / urine / feces / bile of all the analytes met the acceptable criterion of ±15%. The extraction efficiencies indicated that recoveries were consistent and reproducible at different concentrations within the acceptable criteria of ±15%. The coefficient of variation of matrix effect in four concentrations was less than 15%, showing that the contributions of matrix effect were equal at different QC concentrations.

**2.5. Stability**

The stabilities of TPL, WA, WFG, WFT, WFD and WFR were evaluated by analyzing

replicates (n=3) of plasma / liver / urine / feces / bile samples at the lowest and highest QC concentrations, respectively. Samples would experience different conditions: short-term stability storage at room temperature for 4 h, three freeze-thaw cycles at -80°C, post-preparative stability in the autosampler for 24 h, and long-term stability storage at -80°C for 2 weeks. All the samples were analyzed, and the acceptance criteria were set at ±15% (RSD% and RE%). As shown in **Tables S11-S15**, all analytes were stable in rat plasma, liver, urine, feces, and bile samples under the investigated storage conditions anticipated during the routine analysis of these samples.

**3. The correlation between the contents of six key components in TGT and exposures *in vivo***

As shown **in Fig. S7** and **Table S16**, the *in-vivo* exposures of the six TGT components showed a tendency of dose-dependent and were increased correspondingly with the increase of dose.

**4. Evaluation of immunosuppressive activity and hepatotoxicity *in vitro***

The immunosuppressive activities of the six target compounds (TPL, WA, WFG, WFT, WFD, and WFR) on the RAW 264.7 cells and the hepatotoxic effects of the four target compounds (TPL, WFG, WFT, and WFD) on the L02 cells are shown in **Fig. S8** and **Fig. S9**, respectively.

**5.** **HE staining of jejunum and** **ileum samples**

As shown in **Fig. S10,** the jejunum and ileum in M1 and M2 groups exhibited marked decreases in the length and number of villi compared with the Con group, especially in the M2 group.

**
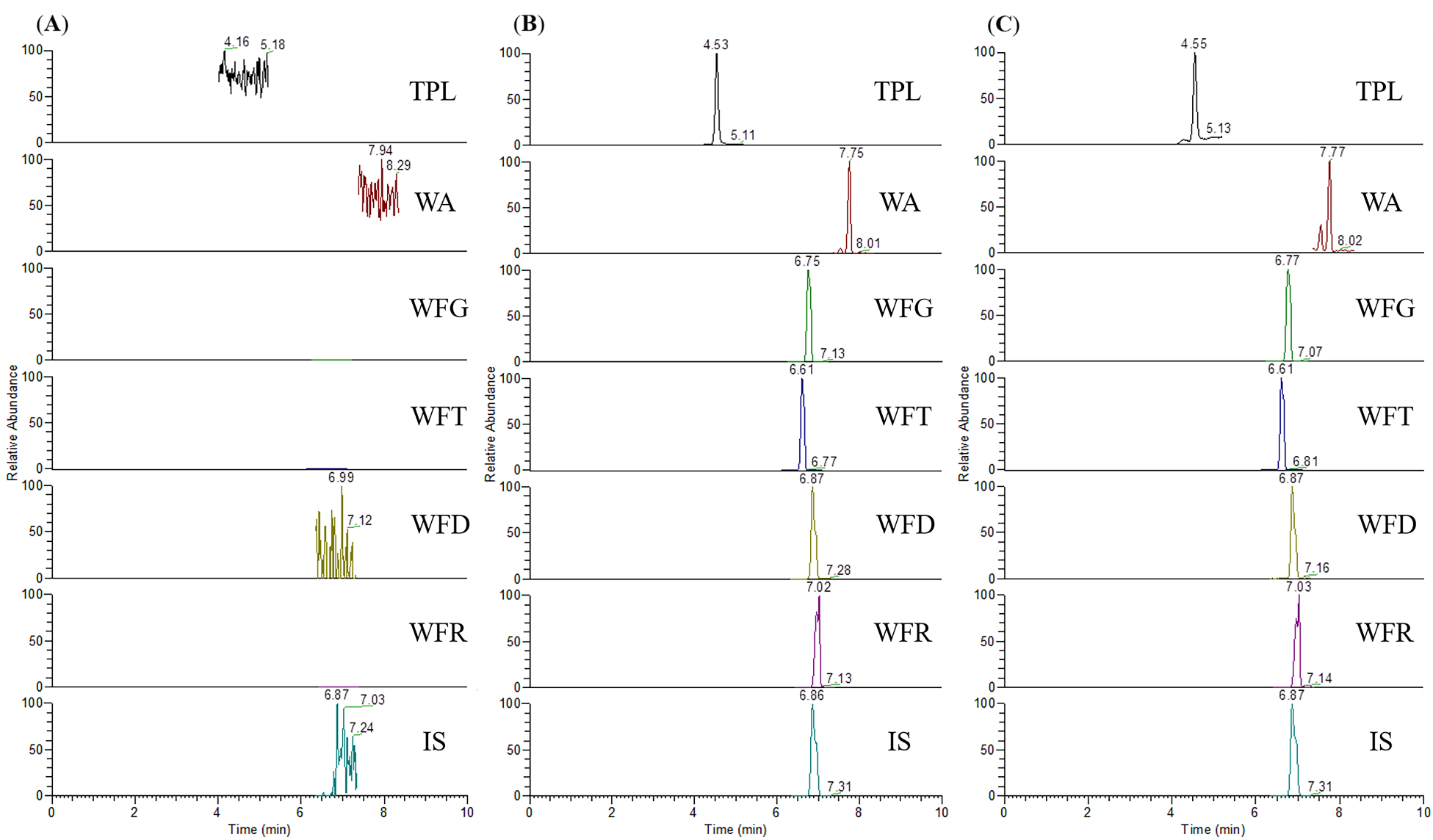
Fig. S2** Representative MRM chromatograms of (**A**) blank plasma, (**B**) blank plasma spiked with TPL, WA, WFG, WFT, WFD, WFR and IS, (**C**) plasma sample obtained from a rat after oral administration of TGT.


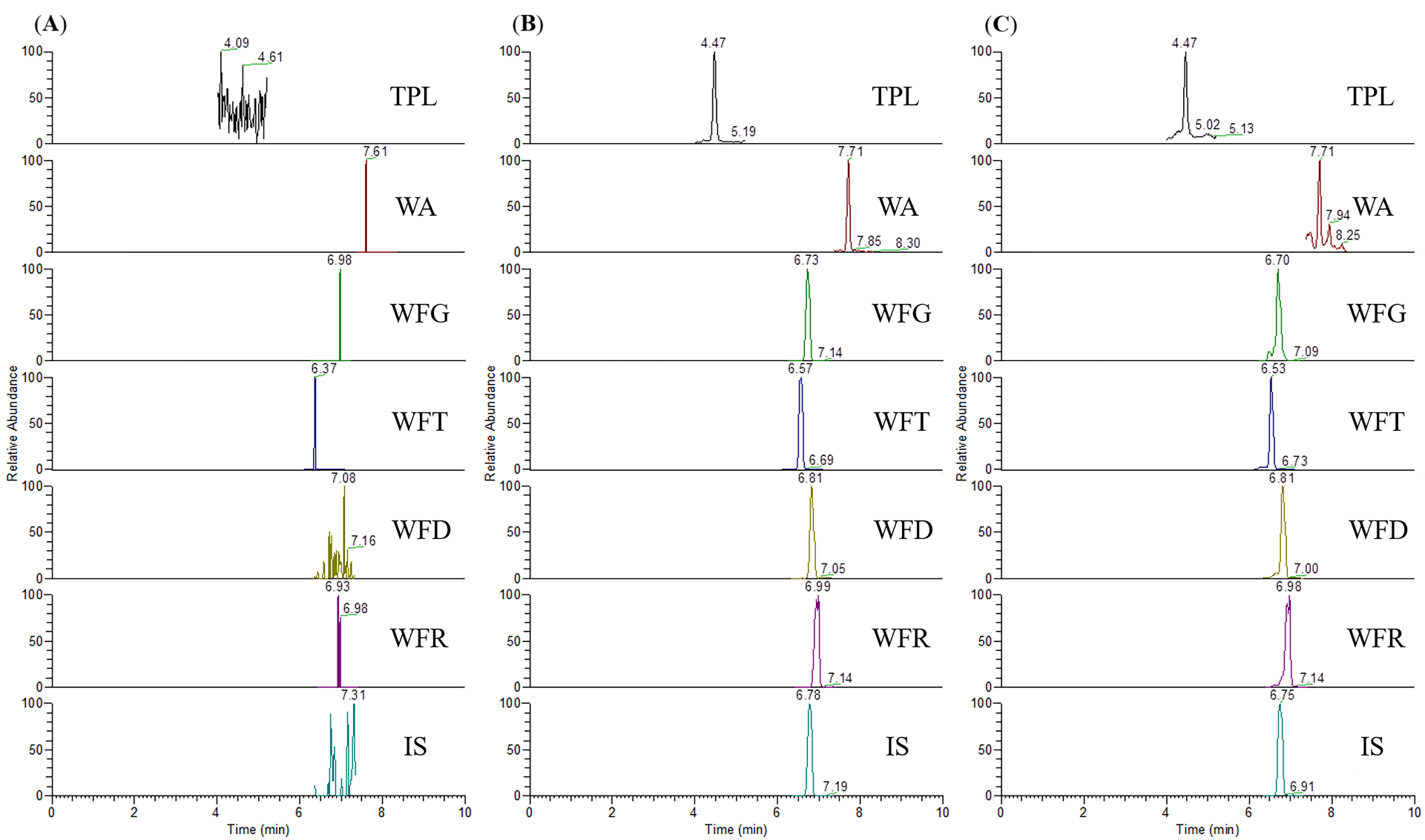
 **Fig. S3** Representative MRM chromatograms of (**A**) blank liver, (**B**) blank liver spiked with TPL, WA, WFG, WFT, WFD, WFR and IS, (**C**) liver sample obtained from a rat after oral administration of TGT.

**
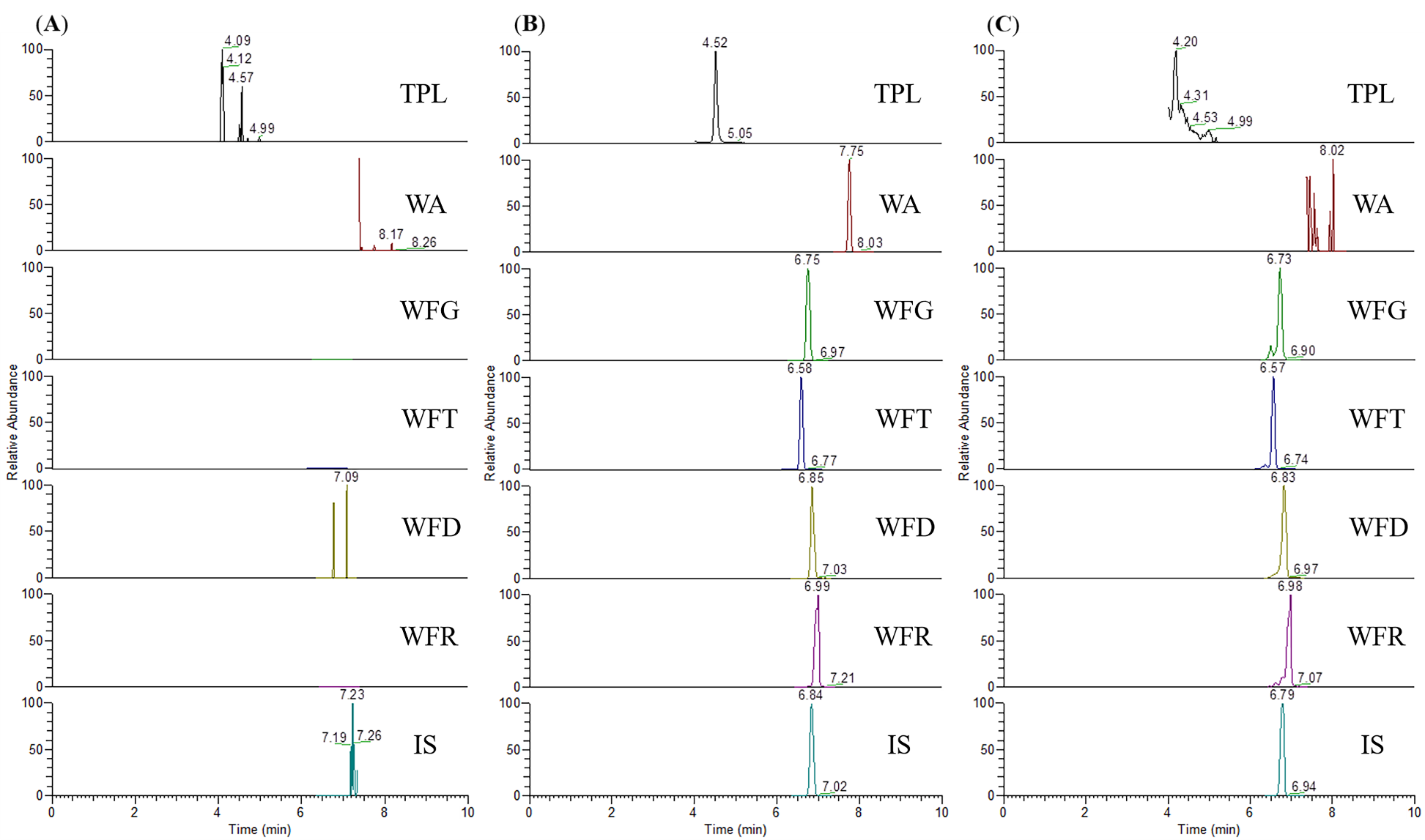
Fig. S4** Representative MRM chromatograms of (**A**) blank urine, (**B**) blank urine spiked with TPL, WA, WFG, WFT, WFD, WFR and IS, (**C**) urine sample obtained from a rat after oral administration of TGT.


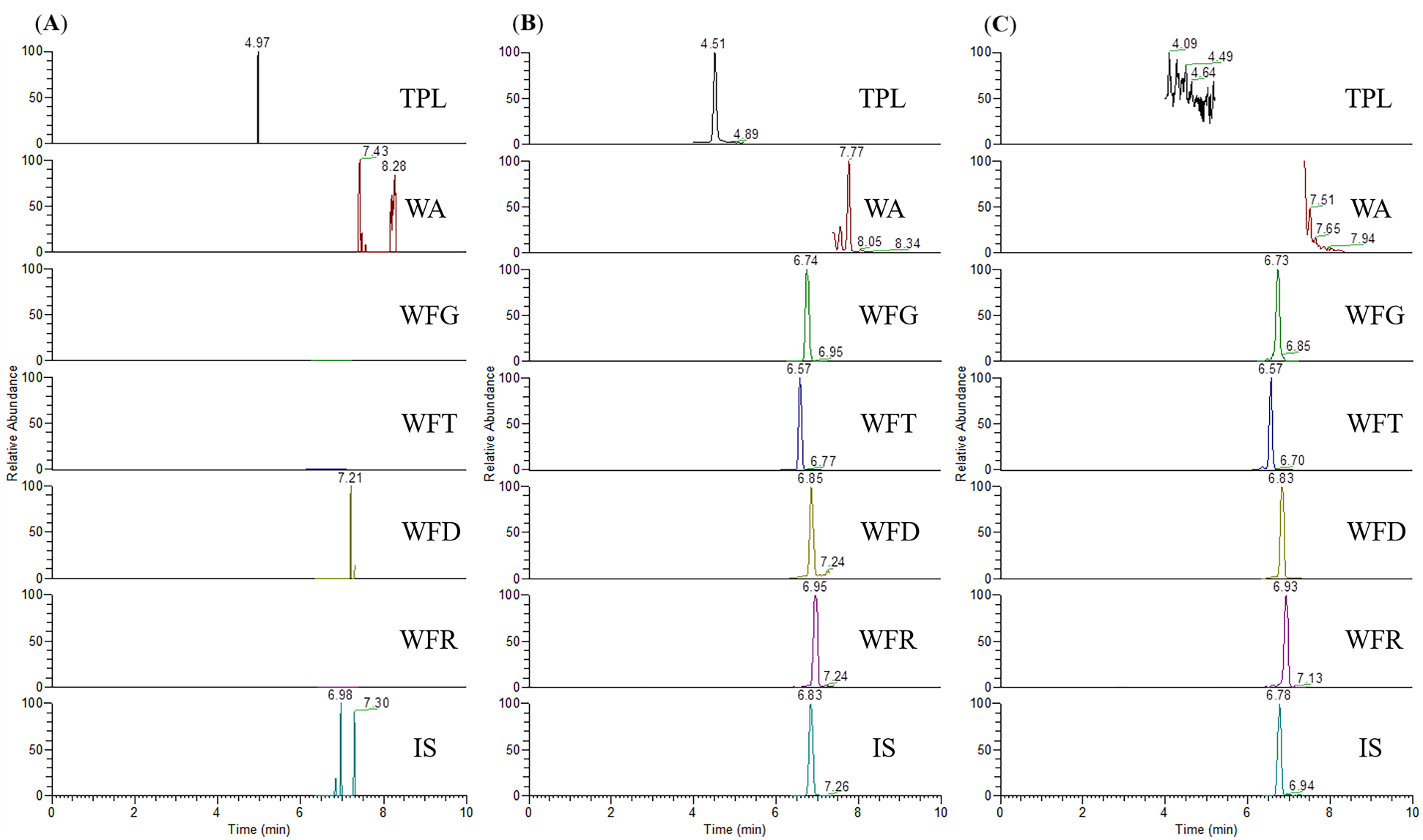
 **Fig. S5** Representative MRM chromatograms of (**A**) blank feces, (**B**) blank feces spiked with TPL, WA, WFG, WFT, WFD, WFR and IS, (**C**) feces sample obtained from a rat after oral administration of TGT.

**
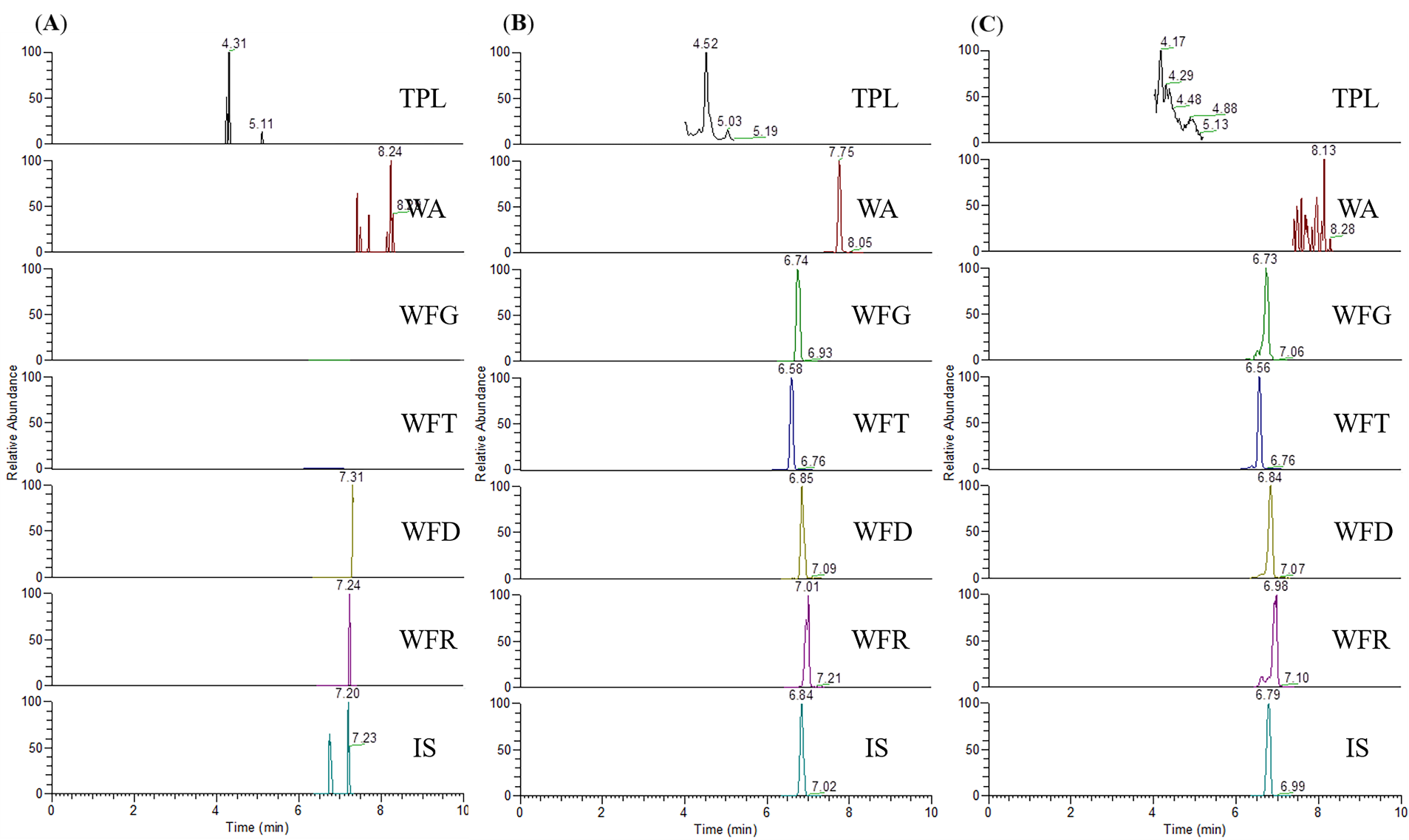
Fig. S6** Representative MRM chromatograms of (**A**) blank bile, (**B**) blank bile spiked with TPL, WA, WFG, WFT, WFD, WFR and IS, (**C**) bile sample obtained from a rat after

oral administration of TGT.


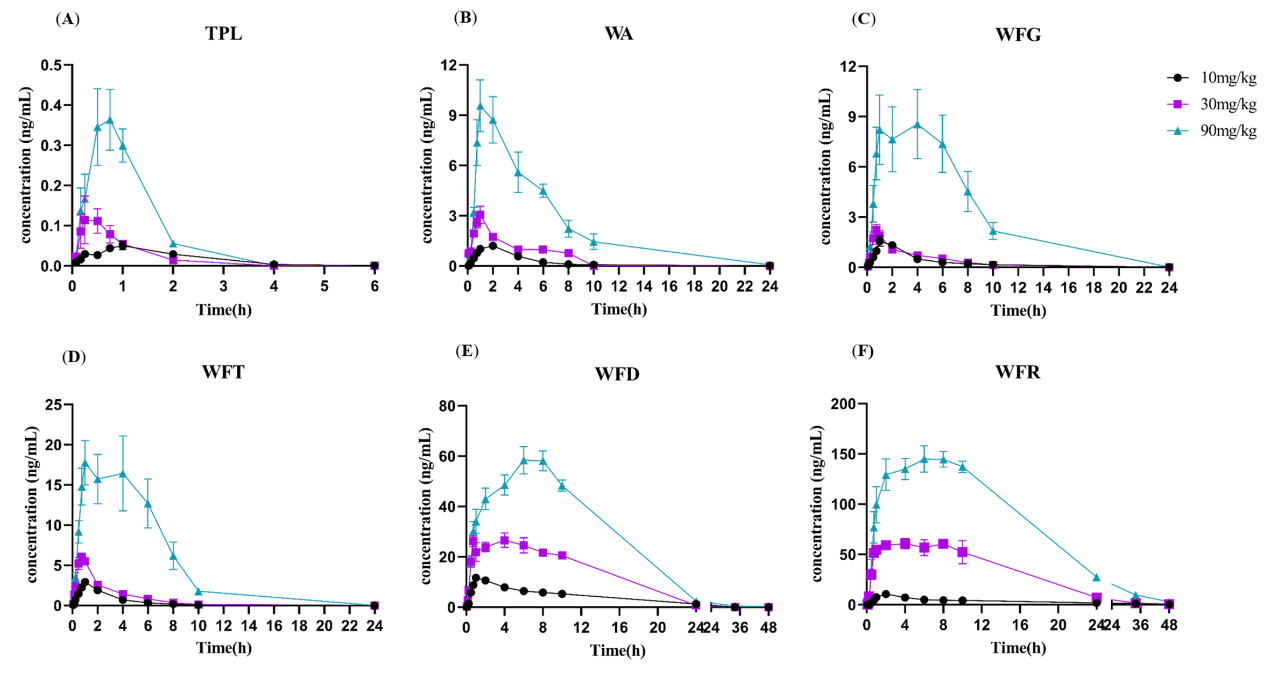
**Fig. S7** Plasma concentration–time profiles of the six key TGT components in the normal rats after a single TGT oral gavage dose of 10 mg/kg, 30 mg/kg or 90 mg/kg (mean ± SD, n = 7). TPL (**A**), WA (**B**), WFG (**C**), WFT (**D**), WFD (**E**), WFR (**F**).


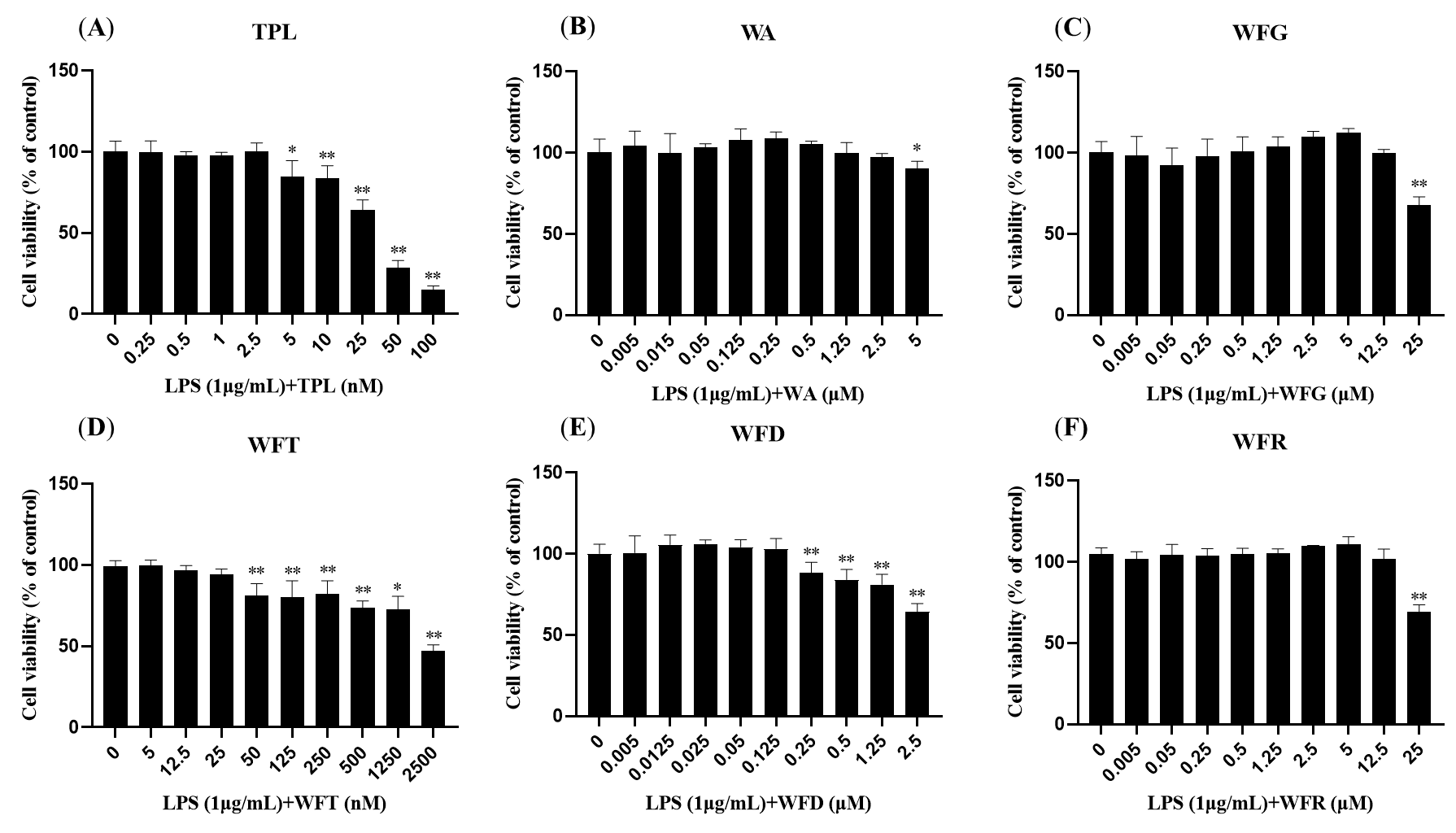
**Fig. S8** The immunosuppressive activities of the six target compounds in LPS-stimulated RAW264.7 cells (mean ± SD, n=6). * p < 0.05, ** p < 0.01 vs. LPS group. TPL(**A**), WA (**B**), WFG (**C**), WFT (**D**), WFD (**E**), WFR (**F)**

**
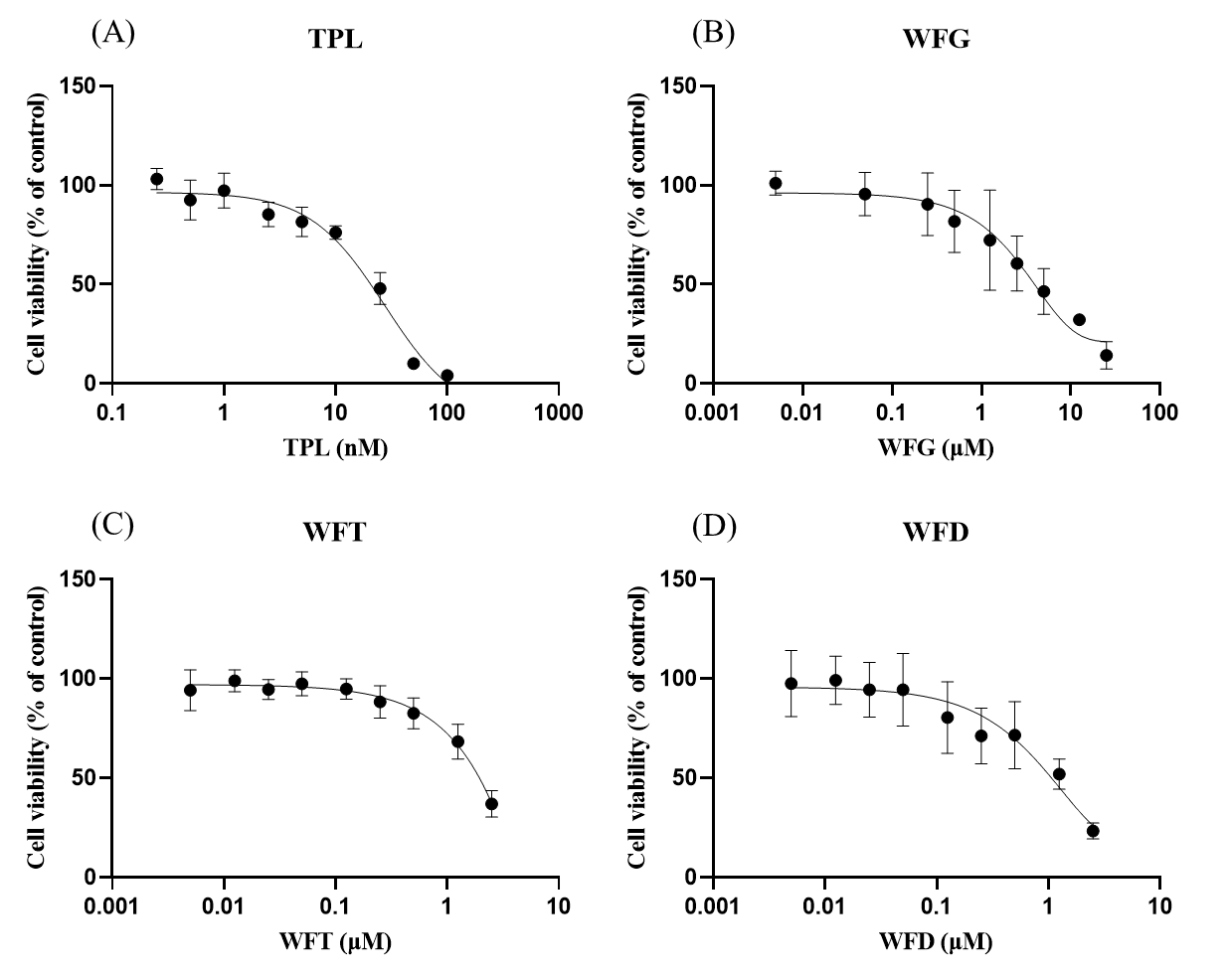
Fig. S9** The hepatotoxic effects of the four target compounds in L02 cells (mean ± SD, n = 6). TPL(**A**), WFG (**B**), WFT (**C**), WFD (**D**).


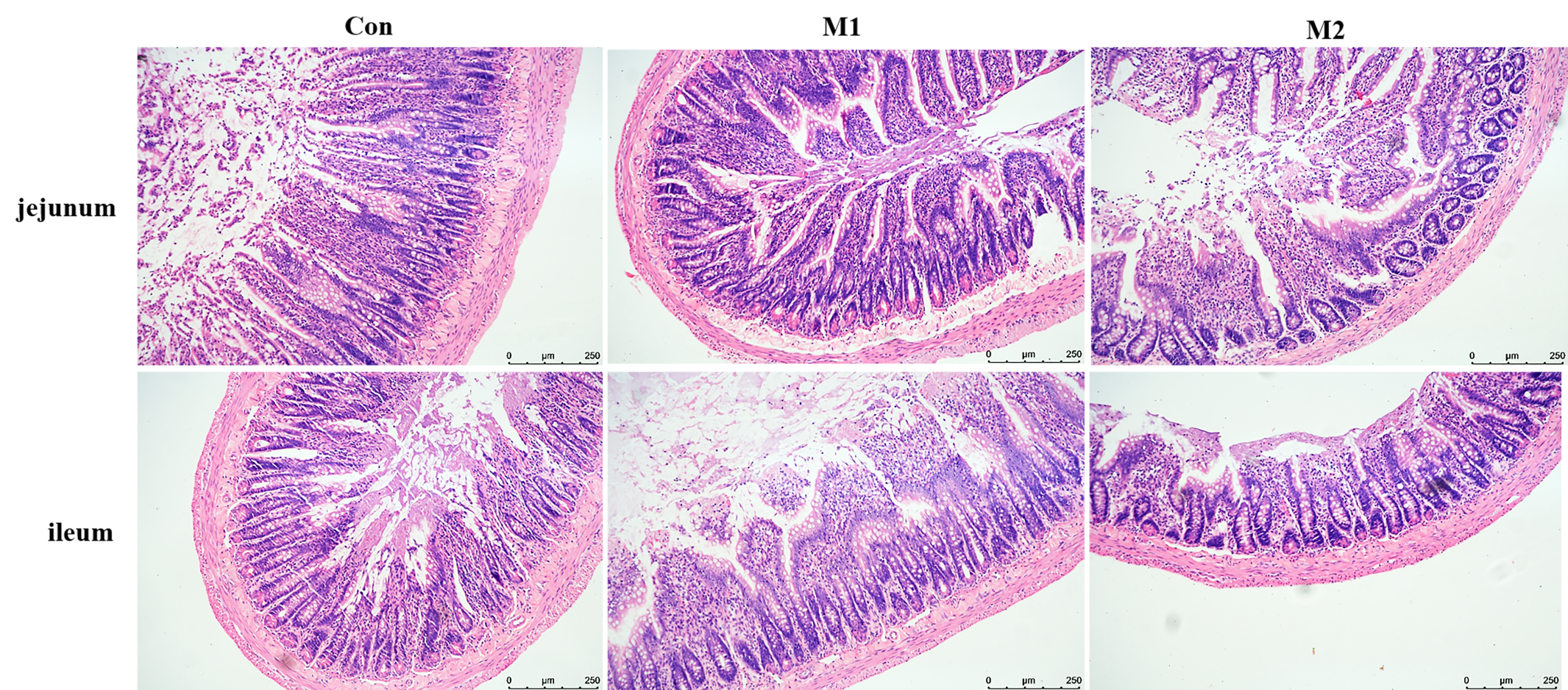


**Fig. S10** HE staining of jejunum and ileum in Con, M1 and M2 groups at the end of the 4th week after the first Adriamycin injection.

| **Table S1** The calibration curves and LLOQs of TPL, WA, WFG, WFT, WFD and WFR in rat plasma | | | | |
| --- | --- | --- | --- | --- |
| Analytes | Regression equation | Linear range (ng/ml) | R^2^ | LLOQ (ng/ml) |
| TPL | Y=0.003061 X + 0.0003122 | 0.02-100 | 0.9964 | 0.02 |
| WA | Y=0.01047 X + 0.00001181 | 0.05-100 | 0.9936 | 0.05 |
| WFG | Y=0.009559 X + 0.00005834 | 0.02-100 | 0.9922 | 0.02 |
| WFT | Y=0.01473 X + 0.0001134 | 0.02-100 | 0.9907 | 0.02 |
| WFD | Y=0.007078 X + 0.00004787 | 0.02-100 | 0.9935 | 0.02 |
| WDR | Y=0.005162 X + 0.00002531 | 0.02-100 | 0.9880 | 0.02 |

| **Table S2** The calibration curves and LLOQs of TPL, WA, WFG, WFT, WFD and WFR in rat liver | | | | |
| --- | --- | --- | --- | --- |
| Analytes | Regression equation | Linear range (ng/ml) | R^2^ | LLOQ (ng/ml) |
| TPL | Y=0.009004 X + 0.0002771 | 0.1-100 | 0.9852 | 0.1 |
| WA | Y=0.005086 X + 0.0001634 | 0.1-100 | 0.9882 | 0.1 |
| WFG | Y=0.006283 X - 0.000142 | 0.1-100 | 0.9882 | 0.1 |
| WFT | Y=0.008751 X - 0.0001103 | 0.1-100 | 0.9894 | 0.1 |
| WFD | Y=0.005112 X - 0.00003532 | 0.1-100 | 0.9882 | 0.1 |
| WDR | Y=0.001115 X + 0.00006796 | 0.1-100 | 0.9915 | 0.1 |

| **Table S3** The calibration curves and LLOQs of TPL, WA, WFG, WFT, WFD and WFR in rat urine | | | | |
| --- | --- | --- | --- | --- |
| Analytes | Regression equation | Linear range (ng/ml) | R^2^ | LLOQ (ng/ml) |
| TPL | Y=0.0008266 X - 0.0002887 | 0.5-100 | 0.9918 | 0.5 |
| WA | Y=0.000244 X - 0.0001897 | 0.2-100 | 0.9965 | 0.2 |
| WFG | Y=0.01717 X - 0.00003235 | 0.05-100 | 0.9924 | 0.05 |
| WFT | Y=0.01278 X - 0.000007884 | 0.05-100 | 0.9907 | 0.05 |
| WFD | Y=0.007982 X + 0.0001576 | 0.05-100 | 0.9954 | 0.05 |
| WDR | Y=0.01061 X - 0.0001948 | 0.05-100 | 0.9923 | 0.05 |

| **Table S4** The calibration curves and LLOQs of TPL, WA, WFG, WFT, WFD and WFR in rat feces | | | | |
| --- | --- | --- | --- | --- |
| Analytes | Regression equation | Linear range (ng/ml) | R^2^ | LLOQ (ng/ml) |
| TPL | Y=0.001004 X + 0.001912 | 0.5-100 | 0.9855 | 0.5 |
| WA | Y=0.0002267 X - 0.0001194 | 0.5-100 | 0.9982 | 0.5 |
| WFG | Y=0.02112 X + 0.002491 | 0.5-100 | 0.9966 | 0.5 |
| WFT | Y=0.01572 X + 0.002123 | 0.5-100 | 0.9958 | 0.5 |
| WFD | Y=0.01184 X + 0.002304 | 0.5-100 | 0.9977 | 0.5 |
| WDR | Y=0.01214 X + 0.001014 | 0.5-100 | 0.9979 | 0.5 |

| **Table S5** The calibration curves and LLOQs of TPL, WA, WFG, WFT, WFD and WFR in rat bile | | | | |
| --- | --- | --- | --- | --- |
| Analytes | Regression equation | Linear range (ng/ml) | R^2^ | LLOQ (ng/ml) |
| TPL | Y=0.02007 X - 0.001894 | 0.2-100 | 0.9841 | 0.2 |
| WA | Y=0.03013 X - 0.01021 | 0.2-100 | 0.9901 | 0.2 |
| WFG | Y=0.01891 X + 0.0004872 | 0.2-100 | 0.9929 | 0.2 |
| WFT | Y=0.0165 X + 0.003306 | 0.2-100 | 0.9888 | 0.2 |
| WFD | Y=0.01496 X + 0.0004061 | 0.2-100 | 0.9879 | 0.2 |
| WDR | Y=0.004394 X + 0.000384 | 0.2-100 | 0.9891 | 0.2 |

| **Table S6** Precision, accuracy, recovery and matrix effect for analysis of TPL, WA, WFG, WFT, WFD and WFR in rat plasma (n = 5). | | | | | | | |
| --- | --- | --- | --- | --- | --- | --- | --- |
| Analytes | Added concentration | Precision (%, RSD) |  | Accuracy (%, RE) |  | Recovery | Matrix Effect |
|  | (ng/mL) | Intra-day (n=5) | Inter-day (n=3) | Intra-day (n=5) | Inter-day (n=3) | (%, mean ± SD, RSD) | (%, mean ± SD, RSD) |
| TPL | 0.05 | 10.9 | 7.85 | -6.00 | -3.73 | 90.4 ± 8.13, 9.00 | 90.7 ± 4.66, 5.14 |
|  | 2.5 | 2.21 | 7.10 | -11.5 | -3.36 | 83.8 ± 2.31, 2.76 | 112 ± 1.83, 1.64 |
|  | 25 | 4.43 | 5.07 | -3.89 | 0.870 | 75.4 ± 2.30, 3.05 | 111 ± 1.92, 1.73 |
|  | 80 | 1.34 | 2.99 | 2.84 | 4.79 | 77.6 ± 1.69, 2.17 | 106 ± 3.44, 3.24 |
| WA | 0.1 | 5.21 | 5.45 | 6.00 | 1.00 | 71.1 ± 7.95, 11.2 | 52. 5 ± 2.70, 5.15 |
|  | 2.5 | 2.18 | 3.84 | -1.38 | 0.100 | 67.9 ± 8.03, 11.8 | 50.0 ± 5.11, 10.2 |
|  | 25 | 1.27 | 5.54 | -1.10 | -3.12 | 70.8 ± 8.21, 11.6 | 47.2 ± 3.82, 8.10 |
|  | 80 | 1.38 | 4.25 | 10.3 | 6.83 | 71.3 ± 9.87, 13.8 | 43.1 ± 0.930, 2.15 |
| WFG | 0.05 | 3.00 | 7.45 | 1.20 | 0.930 | 83.5 ± 8.32, 9.96 | 49.2 ± 1.81, 3.67 |
|  | 2.5 | 1.64 | 6.59 | -8.54 | -9.49 | 83.6 ± 1.46, 1.75 | 50.7 ± 1.80, 3.54 |
|  | 25 | 3.10 | 6.47 | 1.29 | 0.200 | 79.3 ± 2.04, 2.57 | 49.5 ± 0.890, 1.80 |
|  | 80 | 0.990 | 2.35 | 13.2 | 10.9 | 81.7 ± 2.81, 3.44 | 49.0 ± 0.900, 1.83 |
| WFT | 0.05 | 3.90 | 4.74 | -4.00 | -1.14 | 89.0 ± 3.88, 4.36 | 61.9 ± 2.68, 4.33 |
|  | 2.5 | 1.93 | 13.8 | -5.24 | -5.25 | 86.0 ± 1.30, 1.51 | 62.5 ± 5.40, 8.63 |
|  | 25 | 2.58 | 12.5 | 8.87 | 4.37 | 80.1 ± 2.58, 3.22 | 60.8 ± 0.590, 0.970 |
|  | 80 | 2.50 | 8.46 | 11.4 | 5.04 | 82.6 ± 0.490, 0.600 | 60.1 ± 0.760, 1.26 |
| WFD | 0.05 | 4.35 | 10.1 | 0.800 | 1.69 | 91.8 ± 2.99, 3.26 | 54.3 ± 7.78, 14.3 |
|  | 2.5 | 1.60 | 8.79 | 0.0400 | -5.49 | 86.4 ± 2.67, 3.09 | 53.6 ± 4.42, 8.24 |
|  | 25 | 3.96 | 9.50 | 4.59 | -0.540 | 81.6 ± 1.19, 1.46 | 50.1 ± 1.08, 2.15 |
|  | 80 | 1.97 | 3.30 | 11.4 | 8.31 | 85.0 ± 1.49, 1.76 | 51.5 ± 2.76, 5.36 |
| WFR | 0.05 | 4.95 | 12.7 | 9.20 | 6.15 | 80.7 ± 11.5, 14.2 | 55.1 ± 1.09, 1.98 |
|  | 2.5 | 1.78 | 3.05 | 2.45 | 0.690 | 82.5 ± 3.98, 4.83 | 52.1 ± 3.06, 5.88 |
|  | 25 | 5.31 | 4.88 | 3.38 | 0.360 | 83.1 ± 3.17, 3.82 | 49.4 ± 0.790, 1.60 |
|  | 80 | 2.96 | 3.06 | 5.39 | 5.18 | 81.7 ± 5.06, 6.20 | 50.0 ± 1.36, 2.72 |

| **Table S7** Precision, accuracy, recovery and matrix effect for analysis of TPL, WA, WFG, WFT, WFD and WFR in rat liver (n = 5). | | | | | | | |
| --- | --- | --- | --- | --- | --- | --- | --- |
| Analytes | Added concentration | Precision (%, RSD) |  | Accuracy (%, RE) |  | Recovery | Matrix Effect |
|  | (ng/mL) | Intra-day (n=5) | Inter-day (n=3) | Intra-day (n=5) | Inter-day (n=3) | (%, mean ± SD, RSD) | (%, mean ± SD, RSD) |
| TPL | 0.2 | 3.78 | 6.15 | -0.700 | -1.07 | 81.7 ± 3.28, 4.02 | 112 ± 2.07, 1.86 |
|  | 2.5 | 2.28 | 4.97 | -0.0600 | -3.03 | 85.6 ± 4.53, 5.29 | 100 ± 2.80, 2.80 |
|  | 25 | 2.86 | 4.62 | 10.3 | 7.65 | 81.3 ± 5.93, 7.29 | 101 ± 1.83, 1.80 |
|  | 80 | 2.13 | 3.63 | 9.48 | 8.70 | 83.5 ± 2.63, 3.15 | 98.5 ± 2.11, 2.14 |
| WA | 0.2 | 7.10 | 5.77 | 0.700 | 3.07 | 81.0 ± 10.3, 12.8 | 96.0 ± 14.2, 14.8 |
|  | 2.5 | 2.53 | 5.65 | -1.36 | -7.01 | 82.0 ± 3.33, 4.06 | 92.5 ± 2.94, 3.18 |
|  | 25 | 4.22 | 8.71 | 5.21 | -4.67 | 81.2 ± 5.82, 7.17 | 92.5 ± 5.06, 5.48 |
|  | 80 | 1.73 | 4.90 | 8.46 | 3.65 | 80.7 ± 3.84, 4.32 | 90.7 ± 6.14, 6.77 |
| WFG | 0.2 | 4.19 | 3.73 | 5.20 | 4.73 | 78.4 ± 3.47, 4.42 | 105 ± 4.84, 4.60 |
|  | 2.5 | 2.50 | 3.82 | -6.50 | -7.00 | 79.9 ± 2.83, 3.54 | 110 ± 2.07, 1.89 |
|  | 25 | 1.32 | 4.27 | 8.49 | 3.79 | 78.3 ± 3.57, 4.55 | 113 ±3.18, 2.81 |
|  | 80 | 1.49 | 2.52 | 10.5 | 8.96 | 76.2 ± 2.05, 2.69 | 111 ± 2.98, 2.69 |
| WFT | 0.2 | 2.33 | 3.25 | 4.40 | 3.60 | 77.5 ± 5.15, 6.65 | 121 ± 5.19, 4.30 |
|  | 2.5 | 1.63 | 5.33 | -4.21 | -5.44 | 80.9 ± 4.12, 5.09 | 119 ± 3.70, 3.12 |
|  | 25 | 1.44 | 4.35 | 10.6 | 6.76 | 80. 4 ± 4.90, 6.10 | 123 ± 2.47, 2.01 |
|  | 80 | 1.13 | 4.69 | 11.9 | 7.44 | 78.3 ± 2.12, 2.71 | 120 ± 4.24, 3.55 |
| WFD | 0.2 | 1.96 | 3.84 | 2.90 | 1.67 | 79.1 ± 3.19, 4.04 | 92.5 ± 3.83, 4.14 |
|  | 2.5 | 2.28 | 6.83 | 3.64 | -0.860 | 82.7 ± 4.10, 4.95 | 89.7 ± 6.80, 7.59 |
|  | 25 | 2.80 | 4.69 | 6.47 | 3.71 | 83.9 ± 8.57, 10.2 | 92.4 ± 9.18, 9.93 |
|  | 80 | 2.57 | 3.02 | 7.85 | 7.13 | 83.5 ± 3.91, 4.68 | 92.1 ± 7.73, 8.39 |
| WFR | 0.2 | 2.63 | 9.17 | -6.60 | 1.17 | 79.3 ± 5.98, 7.54 | 76.0 ± 7.15, 9.41 |
|  | 2.5 | 2.88 | 6.45 | 1.14 | -0.290 | 78.6 ± 2.46, 3.13 | 77.0 ± 5.59, 7.25 |
|  | 25 | 4.20 | 4.94 | 2.98 | -0.900 | 81.5 ± 8.77, 10.8 | 79.5 ± 7.31, 9.18 |
|  | 80 | 3.32 | 4.19 | 8.93 | 6.45 | 82.0 ± 4.62, 5.64 | 78.3 ± 7.76, 9.92 |

| **Table S8** Precision, accuracy, recovery and matrix effect for analysis of TPL, WA, WFG, WFT, WFD and WFR in rat urine (n = 5). | | | | | | | |
| --- | --- | --- | --- | --- | --- | --- | --- |
| Analytes | Added concentration | Precision (%, RSD) |  | Accuracy (%, RE) |  | Recovery | Matrix Effect |
|  | (ng/mL) | Intra-day (n=5) | Inter-day (n=3) | Intra-day (n=5) | Inter-day (n=3) | (%, mean ± SD, RSD) | (%, mean ± SD, RSD) |
| TPL | 1 | 2.54 | 4.69 | -4.92 | -0.650 | 87.7 ± 3.80, 4.33 | 63.4 ± 1.16, 1.82 |
|  | 2.5 | 2.99 | 5.40 | -6.82 | -1.66 | 83.3 ± 2.65, 3.18 | 76.9 ± 2.28, 2.96 |
|  | 25 | 3.67 | 4.64 | -2.28 | 3.17 | 81.6 ± 2.09, 2.56 | 64.1 ± 0.680, 1.07 |
|  | 80 | 2.97 | 4.00 | 5.08 | 6.06 | 88.7 ± 3.27, 3.68 | 63.8 ± 1.50, 2.35 |
| WA | 0.5 | 2.88 | 6.09 | -5.68 | 1.97 | 85.4 ± 4.35, 5.09 | 99.9 ± 5.74, 5.75 |
|  | 2.5 | 1.84 | 5.60 | -7.66 | -0.680 | 83.3 ± 1.43, 1.72 | 99.6 ± 6.10, 6.13 |
|  | 25 | 2.75 | 4.80 | -3.66 | 1.31 | 84.5 ± 3.77, 4.46 | 103 ± 2.55, 2.47 |
|  | 80 | 3.73 | 4.23 | 3.85 | 5.55 | 88.2 ± 4.08, 4.63 | 105 ± 4.88, 4.63 |
| WFG | 0.1 | 2.93 | 5.54 | -1.60 | 1.33 | 82.4 ± 6.38, 7.74 | 114 ± 5.82, 5.10 |
|  | 2.5 | 2.74 | 3.65 | -4.44 | 0.0300 | 82.7 ± 1.27, 1.53 | 103 ± 1.47, 1.42 |
|  | 25 | 0.630 | 2.40 | 9.15 | 6.39 | 85.2 ± 1.22, 1.43 | 100 ± 1.79, 1.80 |
|  | 80 | 2.30 | 3.05 | 6.46 | 7.66 | 83.7 ± 2.03, 2.43 | 102 ± 3.18, 3.11 |
| WFT | 0.1 | 3.05 | 7.46 | -6.20 | 3.40 | 84. 9 ± 5.17, 6.08 | 142 ± 3.38, 2.39 |
|  | 2.5 | 1.17 | 5.22 | -6.97 | -0.920 | 84.1 ± 1.33, 1.58 | 136 ± 2.66, 1.97 |
|  | 25 | 1.60 | 2.09 | 5.72 | 5.85 | 84.2 ± 0.880, 1.05 | 136 ± 1.86, 1.36 |
|  | 80 | 2.60 | 3.43 | 7.84 | 6.71 | 83.9 ± 2.40, 2.86 | 138 ± 3.82, 2.78 |
| WFD | 0.1 | 7.33 | 5.74 | -3.00 | 1.00 | 80.6 ± 4.52, 5.61 | 115 ± 2.57, 2.24 |
|  | 2.5 | 4.52 | 5.50 | -5.30 | 1.03 | 82.7 ± 4.91, 5.94 | 106 ± 1.84, 1.73 |
|  | 25 | 3.46 | 2.76 | 5.21 | 4.17 | 86.3 ± 3.87, 4.48 | 99.2 ± 4.92, 4.96 |
|  | 80 | 3.51 | 2.84 | 5.43 | 6.94 | 86.3 ± 3.15, 3.65 | 109 ± 8.51, 7.79 |
| WFR | 0.1 | 9.19 | 13.2 | -3.20 | -0.670 | 87.4 ± 8.31, 9.50 | 91.8 ± 5.59, 6.09 |
|  | 2.5 | 5.76 | 4.54 | -2.97 | 1.37 | 82.1 ± 5.28, 6.43 | 88.2 ± 2.57, 2.91 |
|  | 25 | 4.60 | 3.46 | -0.840 | 1.04 | 86.8 ± 4.84, 5.58 | 86.9 ± 2.24, 2.58 |
|  | 80 | 2.41 | 3.62 | 3.32 | 1.74 | 87.7 ± 3.44, 3.93 | 90.5 ± 7.58, 8.38 |

| **Table S9** Precision, accuracy, recovery and matrix effect for analysis of TPL, WA, WFG, WFT, WFD and WFR in rat feces (n = 5). | | | | | | | |
| --- | --- | --- | --- | --- | --- | --- | --- |
| Analytes | Added concentration | Precision (%, RSD) |  | Accuracy (%, RE) |  | Recovery | Matrix Effect |
|  | (ng/mL) | Intra-day (n=5) | Inter-day (n=3) | Intra-day (n=5) | Inter-day (n=3) | (%, mean ± SD, RSD) | (%, mean ± SD, RSD) |
| TPL | 1 | 8.29 | 6.82 | 0.0200 | 0.470 | 103 ± 11.4, 11.1 | 46.4 ± 2.14, 4.61 |
|  | 2.5 | 6.95 | 5.39 | 2.15 | 1.28 | 103 ± 10.5, 10.2 | 43.7 ± 2.83, 6.49 |
|  | 25 | 2.88 | 6.44 | -4.60 | 0.520 | 93.5 ± 4.35, 4.66 | 43.2 ± 2.97, 6.88 |
|  | 80 | 1.83 | 7.02 | -9.68 | -3.39 | 96.2 ± 10.7, 11.1 | 43.6 ± 1.52, 3.49 |
| WA | 1 | 4.99 | 7.02 | -3.58 | -2.16 | 83.6 ± 6.06, 7.25 | 24.8 ± 2.21, 8.91 |
|  | 2.5 | 3.07 | 5.17 | 3.87 | 2.75 | 89.4 ± 4.75, 5.31 | 15.1 ± 1.89, 12.6 |
|  | 25 | 7.17 | 6.25 | 2.59 | -2.48 | 80.3 ± 4.02, 5.00 | 12.3 ± 1.33, 10.8 |
|  | 80 | 2.67 | 6.76 | 7.03 | 2.74 | 79.5 ± 5.98, 7.53 | 14.7 ± 4.43, 30.2 |
| WFG | 1 | 6.35 | 7.33 | -8.82 | -2.05 | 81.6 ± 7.80, 9.56 | 50.0 ± 1.24, 2.47 |
|  | 2.5 | 1.72 | 6.60 | -1.66 | -3.35 | 86.5 ± 1.34, 1.55 | 45.6 ± 0.940, 2.07 |
|  | 25 | 4.20 | 3.41 | 5.05 | 3.56 | 81.0 ± 1.12, 1.39 | 40.2 ± 1.11, 2.77 |
|  | 80 | 1.64 | 3.70 | 3.68 | 4.87 | 80.5 ± 7.51, 9.33 | 40.1 ± 2.83, 7.05 |
| WFT | 1 | 0.650 | 12.2 | -12.6 | -1.67 | 87.3 ± 3.19, 3.65 | 79.9 ± 2.05, 2.56 |
|  | 2.5 | 3.12 | 12.5 | -12.0 | -9.51 | 83.7 ± 11.4, 13.6 | 75.6 ± 1.90, 2.51 |
|  | 25 | 4.66 | 6.44 | -7.77 | -2.39 | 78.2 ± 10.7, 13.7 | 70.0 ± 1.49, 2.13 |
|  | 80 | 4.81 | 3.84 | 6.45 | 5.98 | 83.9 ± 5.36, 6.39 | 67.9 ± 3.59, 5.28 |
| WFD | 1 | 6.53 | 8.20 | -11.2 | -5.21 | 74.1 ± 11.2, 15.1 | 44.4 ± 1.77, 3.98 |
|  | 2.5 | 1.91 | 9.68 | -8.74 | -9.28 | 78.6 ± 11.3, 14.3 | 41.5 ±0.910, 2.20 |
|  | 25 | 2.19 | 5.69 | -6.04 | -1.74 | 72.9 ± 9.70, 13.3 | 37. 8 ± 0.710, 1.87 |
|  | 80 | 2.68 | 6.21 | 6.31 | 1.24 | 76.5 ± 5.36, 7.01 | 37.9 ± 2.46, 6.50 |
| WFR | 1 | 6.85 | 6.45 | -1.22 | -1.23 | 73.6 ± 10.2, 13.9 | 26.1 ± 2.73, 10.4 |
|  | 2.5 | 2.33 | 7.80 | -0.200 | -1.75 | 77.1 ± 6.84, 8.88 | 24.7 ± 2.73, 11.1 |
|  | 25 | 1.60 | 6.69 | 10.1 | 2.78 | 67.1 ± 9.36, 14.0 | 19.4 ± 0.370, 1.91 |
|  | 80 | 2.74 | 7.56 | 10.1 | 1.97 | 75.1 ± 4.37, 5.82 | 19.3 ± 1.60, 8.25 |

| **Table S10** Precision, accuracy, recovery and matrix effect for analysis of TPL, WA, WFG, WFT, WFD and WFR in rat bile (n = 5). | | | | | | | |
| --- | --- | --- | --- | --- | --- | --- | --- |
| Analytes | Added concentration | Precision (%, RSD) | | Accuracy (%, RE) |  | Recovery | Matrix Effect |
|  | (ng/mL) | Intra-day (n=5) | Inter-day (n=3) | Intra-day (n=5) | Inter-day (n=3) | (%, mean ± SD, RSD) | (%, mean ± SD, RSD) |
| TPL | 0.5 | 4.42 | 4.34 | 11.6 | -5.00 | 93.0 ± 5.83, 6.27 | 75.9 ± 7.08, 6.69 |
|  | 2.5 | 5.96 | 3.20 | 1.70 | 3.34 | 93.1 ± 8.24, 8.86 | 78.6 ± 2.85, 3.22 |
|  | 25 | 5.69 | 7.93 | 3.65 | 1.61 | 87.4 ± 1.57, 1.79 | 65.5 ± 2.37, 3.61 |
|  | 80 | 3.20 | 3.99 | 5.66 | 4.51 | 92.7 ± 1.04, 1.12 | 71.0 ± 2.80, 3.94 |
| WA | 0.5 | 2.23 | 7.19 | -5.76 | 0.640 | 98.2 ± 6.41, 6.52 | 67.7 ± 1.36, 2.01 |
|  | 2.5 | 1.98 | 4.90 | 5.70 | 2.93 | 97.1 ± 6.45, 6.64 | 61.1 ± 3.27, 5.35 |
|  | 25 | 6.33 | 3.02 | 3.40 | -5.69 | 96.9 ± 4.25, 4.39 | 55.7 ± 2.43, 4.37 |
|  | 80 | 2.70 | 7.08 | 9.60 | 4.51 | 101 ± 2.71, 2.69 | 57.0 ± 1.91, 3.35 |
| WFG | 0.5 | 1.38 | 5.98 | 7.20 | -2.20 | 87.6 ± 5.76, 6.58 | 113 ± 0.880, 0.780 |
|  | 2.5 | 4.84 | 2.27 | 5.38 | -3.84 | 84.7 ± 4.27, 5.04 | 110 ± 3.99, 3.63 |
|  | 25 | 4.99 | 2.05 | 11.3 | -1.10 | 82.4 ± 2.89, 3.51 | 107± 5.54, 5.16 |
|  | 80 | 2.21 | 3.22 | 11.5 | 6.58 | 89.7 ± 9.12, 10.2 | 106 ± 13.5, 12.7 |
| WFT | 0.5 | 6.99 | 2.22 | 3.80 | -13.2 | 95.5 ± 10.3, 10.8 | 82.4 ± 5.75, 6.98 |
|  | 2.5 | 7.26 | 5.69 | 5.82 | -2.95 | 88.7 ± 9.42, 10.6 | 79.1± 3.96, 5.01 |
|  | 25 | 7.22 | 6.27 | 11.9 | 2.95 | 91.0 ± 5.14, 5.65 | 76.6 ± 11.2, 14.6 |
|  | 80 | 2.26 | 3.00 | 12.0 | 7.47 | 94.2 ± 0.860, 0.910 | 72.5 ± 4.23, 5.83 |
| WFD | 0.5 | 5.44 | 5.03 | 8.20 | -11.8 | 85.1 ± 9.76, 11.5 | 74.8 ± 3.19, 4.26 |
|  | 2.5 | 7.14 | 3.97 | 2.07 | -2.00 | 84.0 ± 9.04, 10.8 | 73.5 ± 3.67, 4.99 |
|  | 25 | 6.25 | 4.10 | 7.98 | 0.100 | 83.9 ± 9.42, 11.2 | 69.8 ± 10.4, 14.9 |
|  | 80 | 6.34 | 4.55 | 4.84 | 7.75 | 88.3 ± 6.31, 7.14 | 63.8 ± 3.99, 6.25 |
| WFR | 0.5 | 6.18 | 8.14 | 6.40 | 2.60 | 86.9 ± 11.2, 12.9 | 89.2 ± 6.05, 6.78 |
|  | 2.5 | 5.76 | 7.36 | 8.26 | 2.93 | 87.4 ± 11.3, 12.9 | 87.0 ± 3.45, 3.97 |
|  | 25 | 3.22 | 5.88 | 7.88 | -1.46 | 84.4 ± 3.44, 4.07 | 81.2 ± 4.14, 5.10 |
|  | 80 | 5.20 | 8.13 | 7.15 | 3.57 | 88.1 ± 6.60, 7.49 | 82.1 ± 11.2, 13.6 |

| **Table S11** Stability for analysis of TPL, WA, WFG, WFT, WFD and WFR in rat plasma (n = 3). | | | | | | | | | |
| --- | --- | --- | --- | --- | --- | --- | --- | --- | --- |
| Analytes | Added concentration | Room temperature for 4 h | | Freeze-thaw (3times, -80◦C) | | Autosampler for 24 h(-4◦C) | | -80◦C storage for 2 w | |
|  | (ng/mL) | RSD (%) | RE (%) | RSD (%) | RE (%) | RSD (%) | RE (%) | RSD (%) | RE (%) |
| TPL | 0.05 | 10.8 | -5.33 | 6.03 | 1.33 | 11.8 | -0.670 | 9.58 | -1.33 |
|  | 80 | 1.66 | 3.65 | 0.210 | 0.940 | 1.01 | -8.94 | 2.06 | 8.49 |
| WA | 0.1 | 7.07 | -1.00 | 12.4 | -2.67 | 7.62 | -0.670 | 3.31 | -7.67 |
|  | 80 | 1.72 | 7.14 | 2.82 | 9.45 | 1.83 | 3.76 | 6.18 | 5.92 |
| WFG | 0.05 | 5.52 | 10.7 | 3.27 | 6.00 | 2.32 | -0.670 | 11.8 | -0.670 |
|  | 80 | 2.95 | 1.13 | 4.85 | 5.01 | 4.18 | 8.03 | 3.74 | 6.46 |
| WFT | 0.05 | 1.08 | 7.33 | 7.82 | 3.33 | 9.26 | -12.7 | 2.04 | 13.3 |
|  | 80 | 0.730 | 6.96 | 2.00 | 6.48 | 0.510 | 7.89 | 3.44 | 6.45 |
| WFD | 0.05 | 1.04 | 11.3 | 3.95 | 5.33 | 1.08 | 6.67 | 12.4 | -1.33 |
|  | 80 | 3.68 | 0.610 | 8.58 | 4.61 | 3.27 | 4.85 | 3.68 | 3.53 |
| WFR | 0.05 | 9.55 | 3.33 | 1.82 | 10.0 | 12.1 | 4.67 | 12.1 | 5.00 |
|  | 80 | 1.01 | -1.24 | 3.49 | 3.13 | 1.76 | 1.32 | 6.46 | -2.32 |

| **Table S12** Stability for analysis of TPL, WA, WFG, WFT, WFD and WFR in rat liver (n = 3). | | | | | | | | | |
| --- | --- | --- | --- | --- | --- | --- | --- | --- | --- |
| Analytes | Added concentration | Room temperature for 4 h | | Freeze-thaw (3times, -80◦C) | | Autosampler for 24 h(-4◦C) | | -80◦C storage for 2 w | |
|  | (ng/mL) | RSD (%) | RE (%) | RSD (%) | RE (%) | RSD (%) | RE (%) | RSD (%) | RE (%) |
| TPL | 0.2 | 2.87 | 0.670 | 8.91 | 3.00 | 4.90 | 6.00 | 5.42 | -5.83 |
|  | 80 | 3.59 | 8.05 | 2.82 | 11.1 | 3.24 | -4.69 | 5.24 | 4.91 |
| WA | 0.2 | 3.71 | 7.00 | 6.29 | 6.67 | 9.39 | 1.50 | 6.75 | -1.33 |
|  | 80 | 3.99 | 3.62 | 2.37 | 1.40 | 2.64 | 10.9 | 0.370 | 6.70 |
| WFG | 0.2 | 1.88 | 7.67 | 3.85 | 5.83 | 7.07 | 6.17 | 0.790 | 9.00 |
|  | 80 | 1.96 | 11.2 | 0.470 | 8.34 | 3.88 | 7.02 | 0.300 | 6.17 |
| WFT | 0.2 | 1.49 | 7.67 | 1.23 | 7.50 | 5.96 | -3.17 | 2.10 | 4.00 |
|  | 80 | 2.02 | 8.84 | 0.730 | 11.7 | 3.68 | -9.64 | 1.83 | 1.23 |
| WFD | 0.2 | 2.97 | 2.83 | 0.970 | 7.67 | 1.68 | 3.00 | 0.280 | 1.33 |
|  | 80 | 1.25 | 10.7 | 3.45 | 9.61 | 2.18 | 7.33 | 5.10 | 5.76 |
| WFR | 0.2 | 3.74 | 1.00 | 8.44 | -5.17 | 0.860 | 0.500 | 13.6 | 11.5 |
|  | 80 | 3.06 | 5.57 | 3.22 | 3.70 | 2.73 | 9.36 | 2.35 | 5.91 |

| **Table S13** Stability for analysis of TPL, WA, WFG, WFT, WFD and WFR in rat urine (n = 3). | | | | | | | | | |
| --- | --- | --- | --- | --- | --- | --- | --- | --- | --- |
| Analytes | Added concentration | Room temperature for 4 h | | Freeze-thaw (3times, -80◦C) | | Autosampler for 24 h(-4◦C) | | -80◦C storage for 2 w | |
|  | (ng/mL) | RSD (%) | RE (%) | RSD (%) | RE (%) | RSD (%) | RE (%) | RSD (%) | RE (%) |
| TPL | 1 | 10.1 | 0.300 | 5.45 | -4.17 | 2.10 | 2.63 | 8.76 | -0.0700 |
|  | 80 | 2.72 | 10.2 | 2.30 | 11.0 | 3.22 | 7.92 | 0.0500 | -1.77 |
| WA | 0.5 | 6.50 | -2.13 | 4.51 | 1.60 | 3.24 | -0.800 | 1.84 | -9.53 |
|  | 80 | 2.46 | 2.99 | 2.32 | 8.50 | 3.97 | 6.91 | 1.86 | 11.9 |
| WFG | 0.1 | 4.85 | 1.67 | 6.32 | 5.33 | 7.38 | 1.67 | 3.61 | 0.100 |
|  | 80 | 5.30 | 5.63 | 1.26 | 8.95 | 3.99 | 10.5 | 1.56 | 6.71 |
| WFT | 0.1 | 3.24 | 7.00 | 2.36 | 6.67 | 3.23 | 8.67 | 5.34 | -1.00 |
|  | 80 | 5.89 | 4.91 | 1.09 | 8.62 | 2.24 | 6.02 | 2.38 | 11.4 |
| WFD | 0.1 | 9.33 | -3.33 | 4.88 | -6.00 | 2.01 | 3.67 | 5.42 | 1.67 |
|  | 80 | 2.39 | 0.970 | 3.82 | 3.71 | 0.840 | 5.92 | 4.59 | 7.14 |
| WFR | 0.1 | 2.99 | 7.33 | 10.2 | 0.670 | 2.99 | 7.67 | 3.09 | -3.00 |
|  | 80 | 4.19 | -2.94 | 1.92 | 3.26 | 1.94 | -0.360 | 4.17 | 7.09 |

| **Table S14** Stability for analysis of TPL, WA, WFG, WFT, WFD and WFR in rat feces (n = 3). | | | | | | | | | |
| --- | --- | --- | --- | --- | --- | --- | --- | --- | --- |
| Analytes | Added concentration | Room temperature for 4 h | | Freeze-thaw (3times, -80◦C) | | Autosampler for 24 h(-4◦C) | | -80◦C storage for 2 w | |
|  | (ng/mL) | RSD (%) | RE (%) | RSD (%) | RE (%) | RSD (%) | RE (%) | RSD (%) | RE (%) |
| TPL | 1 | 3.93 | 10.5 | 3.96 | -3.63 | 6.46 | -6.90 | 4.80 | -1.27 |
|  | 80 | 7.20 | 5.11 | 3.09 | -2.53 | 2.24 | 6.21 | 3.98 | 4.74 |
| WA | 1 | 7.93 | 0.500 | 13.3 | -5.80 | 1.80 | -0.200 | 8.63 | 3.17 |
|  | 80 | 7.19 | -3.30 | 4.16 | 4.71 | 6.20 | -1.64 | 2.59 | 5.78 |
| WFG | 1 | 0.820 | 12.9 | 0.540 | 12.4 | 1.76 | 13.3 | 0.430 | -5.27 |
|  | 80 | 12.5 | 3.49 | 10.7 | -2.85 | 10.5 | 4.46 | 1.11 | 5.07 |
| WFT | 1 | 4.36 | -14.8 | 7.48 | -10.7 | 12.0 | -11.3 | 8.19 | -9.77 |
|  | 80 | 3.16 | 3.53 | 6.15 | 4.84 | 4.16 | 6.52 | 2.16 | 11.3 |
| WFD | 1 | 3.15 | -13.9 | 2.01 | -14.9 | 11.8 | -8.20 | 4.53 | -1.20 |
|  | 80 | 8.19 | 1.49 | 3.22 | 1.48 | 3.66 | 3.64 | 5.83 | 6.66 |
| WFR | 1 | 2.71 | -2.13 | 3.36 | 10.8 | 8.21 | -5.73 | 6.49 | 2.77 |
|  | 80 | 13.1 | -14.3 | 4.07 | 4.32 | 10.6 | 5.69 | 6.50 | 1.65 |

| **Table S15.** Stability for analysis of TPL, WA, WFG, WFT, WFD and WFR in rat bile (n = 3). | | | | | | | | | |
| --- | --- | --- | --- | --- | --- | --- | --- | --- | --- |
| Analytes | Added concentration | Room temperature for 4 h | | Freeze-thaw (3times, -80◦C) | | Autosampler for 24 h (-4◦C) | | -80◦C storage for 2 w | |
|  | (ng/mL) | RSD (%) | RE (%) | RSD (%) | RE (%) | RSD (%) | RE (%) | RSD (%) | RE (%) |
| TPL | 0.5 | 2.59 | -2.67 | 5.81 | 3.67 | 6.68 | -9.00 | 6.07 | 0.670 |
|  | 80 | 7.68 | 1.33 | 10.0 | -7.31 | 4.54 | 5.16 | 0.960 | 9.14 |
| WA | 0.5 | 0.120 | -5.67 | 7.48 | -3.60 | 6.80 | -6.47 | 4.92 | 1.67 |
|  | 80 | 4.17 | 1.57 | 4.23 | 2.41 | 6.24 | 6.99 | 1.34 | -3.98 |
| WFG | 0.5 | 3.77 | -8.00 | 3.09 | -18.7 | 6.93 | 4.33 | 6.19 | -3.00 |
|  | 80 | 2.37 | 2.27 | 8.70 | 5.02 | 3.59 | 9.31 | 0.210 | 11.7 |
| WFT | 0.5 | 0.650 | -11.7 | 4.37 | -13.3 | 2.97 | -11.0 | 11.3 | -2.67 |
|  | 80 | 3.49 | 5.36 | 5.83 | 4.17 | 3.86 | 6.81 | 2.18 | 11.9 |
| WFD | 0.5 | 1.67 | 4.00 | 1.86 | -7.00 | 2.02 | -1.00 | 3.96 | 1.00 |
|  | 80 | 1.90 | 1.18 | 8.83 | 5.12 | 0.680 | 6.10 | 1.94 | 9.04 |
| WFR | 0.5 | 10.3 | 6.67 | 0.650 | -11.3 | 8.32 | 3.67 | 14.6 | -5.00 |
|  | 80 | 1.78 | -6.70 | 9.29 | -1.24 | 10.8 | 3.38 | 7.46 | 5.00 |

| **Table S16.** Pharmacokinetic parameters of the six key TGT components in the normal rats after a single 10, 30 or 90 mg/kg TGT oral gavage dose (mean ± SD, n=7). | | | | | | | | | | | |
| --- | --- | --- | --- | --- | --- | --- | --- | --- | --- | --- | --- |
| Components | Dose (μg /kg) | C _max_ (ng/mL) | T _max_ (h) | AUC_0-t_ (ng*h/mL) | AUC_0-∞_ (ng*h/mL) | T_1/2_ (h) | Vd (L/kg) | CL (L/h/kg) | MRT_0-t_ (h) | MRT_0-∞_ (h) |  |
| TPL | 5.90 | 0.05 ± 0.03 | 0.92 ± 0.13 | 0.10 ± 0.06 | 0.10 ± 0.03 | 0.73 ± 0.14 | 63.9 ± 28.6 | 59.1 ± 15.6 | 1.26 ± 0.31 | 1.46 ± 0.04 |  |
|  | 17.7 | 0.14 ± 0.11 | 0.50 ± 0.20 | 0.12 ± 0.06 | 0.13 ± 0.08 | 0.60 ± 0.16 | 155 ± 91.0 | 172 ± 94.8 | 0.75 ± 0.09 | 0.98 ± 0.22 |  |
|  | 53.1 | 0.39 ± 0.14 | 0.69 ± 0.24 | 0.43 ± 0.16 | 0.57 ± 0.04 | 0.38 ± 0.02 | 52.0 ± 0.60 | 94.1 ± 6.70 | 0.86 ± 0.09 | 0.89 ± 0.06 |  |
| WA | 25.4 | 1.30 ± 0.26 | 1.57 ± 0.53 | 4.67 ± 0.92 | 4.92 ± 1.01 | 2.11 ± 0.62 | 16.3 ± 6.13 | 5.35 ± 1.12 | 2.99 ± 0.48 | 3.48 ± 0.75 |  |
|  | 76.2 | 2.98 ± 1.14 | 1.11 ± 0.40 | 10.5 ± 2.01 | 25.7 ± 13.8 | 5.25 ± 0.60 | 50.1 ± 22.8 | 3.49 ± 1.23 | 3.21 ± 0.20 | 7.3 ± 0.90 |  |
|  | 228.6 | 9.95 ± 3.04 | 1.25 ± 0.50 | 58.6 ± 8.64 | 59.0 ± 8.44 | 3.52 ± 1.07 | 20.2 ± 8.33 | 3.92 ± 0.53 | 5.06 ± 1.07 | 5.25 ± 1.01 |  |
| WFG | 148 | 1.59 ± 0.76 | 1.29 ± 0.49 | 4.86 ± 1.83 | 5.70 ± 2.26 | 3.53 ± 0.33 | 114 ± 15.6 | 24.4 ± 11.7 | 3.12 ± 0.29 | 4.88 ± 0.52 |  |
|  | 444 | 2.26 ± 0.85 | 0.79 ± 0.09 | 7.13 ± 2.67 | 7.61 ± 2.75 | 2.30 ± 0.38 | 227 ± 122 | 65.8 ± 25.7 | 3.26 ± 0.26 | 3.95 ± 0.50 |  |
|  | 1332 | 9.99 ± 6.08 | 3.57 ± 1.62 | 77.9 ± 45.7 | 77.9 ± 45.7 | 1.89 ± 0.35 | 65.1 ± 44.0 | 23.3 ± 13.9 | 5.66 ± 0.66 | 5.67 ± 0.66 |  |
| WFT | 86.0 | 2.97 ± 1.06 | 0.96 ± 0.09 | 8.64 ± 3.48 | 8.75 ± 3.49 | 2.57 ± 1.11 | 39.7 ± 17.2 | 11.6 ± 5.50 | 2.83 ± 0.62 | 3.01 ± 0.59 |  |
|  | 258 | 6.22 ± 1.39 | 0.79 ± 0.17 | 17.4 ± 4.67 | 17.5 ± 4.78 | 2.53 ± 0.82 | 58.6 ± 26.8 | 15.8 ± 4.80 | 2.90 ± 0.29 | 3.04 ± 0.27 |  |
|  | 774 | 19.5 ± 9.03 | 2.00 ± 1.55 | 126 ± 66.9 | 126 ± 66.9 | 1.66 ± 0.53 | 20.1 ± 14.9 | 7.66 ± 3.67 | 4.65 ± 0.72 | 4.66 ± 0.71 |  |
| WFD | 122 | 12.5 ± 2.01 | 1.43± 0.53 | 137 ± 24.8 | 138 ± 25.4 | 8.24± 1.36 | 10.6 ± 1.49 | 0.91 ± 0.17 | 10.9± 2.01 | 11.2 ± 2.29 |  |
|  | 366 | 29.2 ± 5.43 | 2.39 ± 1.55 | 382 ± 61.8 | 382 ± 61.8 | 3.41 ± 0.26 | 4.78 ± 0.62 | 0.98 ± 0.15 | 7.45 ± 0.41 | 7.46 ± 0.41 |  |
|  | 1098 | 61.7 ± 11.5 | 6.57 ± 1.51 | 852 ± 100 | 854 ± 102 | 4.32 ± 1.36 | 7.91 ± 1.80 | 1.30 ± 0.15 | 8.22 ± 0.60 | 8.35 ± 0.79 |  |
| WFR | 175 | 10.6 ± 1.52 | 1.86 ± 0.38 | 137 ± 20.5 | 141 ± 21.6 | 14.4 ± 1.93 | 26.3 ± 5.96 | 1.26 ± 0.19 | 16.1 ± 1.78 | 18.4 ± 2.18 |  |
|  | 525 | 68.2 ± 7.02 | 3.00 ± 2.00 | 1027 ± 284 | 1033 ± 287 | 5.82 ± 0.56 | 4.49 ± 1.07 | 0.55 ± 0.20 | 8.82 ± 1.61 | 9.06 ± 1.69 |  |
|  | 1575 | 158 ± 26.3 | 5.20 ± 2.28 | 2696 ± 234 | 2726 ± 235 | 6.87 ± 1.13 | 5.78 ± 1.22 | 0.58 ± 0.05 | 10.9 ± 1.09 | 11.4 ± 1.44 |  |
